# Analysis of the Effects of Polymorphism on Pollen Profilin Structural Functionality and the Generation of Conformational, T- and B-Cell Epitopes

**DOI:** 10.1371/journal.pone.0076066

**Published:** 2013-10-17

**Authors:** Jose C. Jimenez-Lopez, María I. Rodríguez-García, Juan D. Alché

**Affiliations:** Department of Biochemistry, Cell and Molecular Biology of plants, Estación Experimental del Zaidín (EEZ), High Council for Scientific Research (CSIC), Granada, Spain; University of South Florida College of Medicine, United States of America

## Abstract

An extensive polymorphism analysis of pollen profilin, a fundamental regulator of the actin cytoskeleton dynamics, has been performed with a major focus in 3D-folding maintenance, changes in the 2-D structural elements, surface residues involved in ligands-profilin interactions and functionality, and the generation of conformational and lineal B- and T-cell epitopes variability.

Our results revealed that while the general fold is conserved among profilins, substantial structural differences were found, particularly affecting the special distribution and length of different 2-D structural elements (i.e. cysteine residues), characteristic loops and coils, and numerous micro-heterogeneities present in fundamental residues directly involved in the interacting motifs, and to some extension these residues nearby to the ligand-interacting areas. Differential changes as result of polymorphism might contribute to generate functional variability among the plethora of profilin isoforms present in the olive pollen from different genetic background (olive cultivars), and between plant species, since biochemical interacting properties and binding affinities to natural ligands may be affected, particularly the interactions with different actin isoforms and phosphoinositides lipids species.

Furthermore, conspicuous variability in lineal and conformational epitopes was found between profilins belonging to the same olive cultivar, and among different cultivars as direct implication of sequences polymorphism. The variability of the residues taking part of IgE-binding epitopes might be the final responsible of the differences in cross-reactivity among olive pollen cultivars, among pollen and plant-derived food allergens, as well as between distantly related pollen species, leading to a variable range of allergy reactions among atopic patients. Identification and analysis of commonly shared and specific epitopes in profilin isoforms is essential to gain knowledge about the interacting surface of these epitopes, and for a better understanding of immune responses, helping design and development of rational and effective immunotherapy strategies for the treatment of allergy diseases.

## Introduction

Profilins are ubiquitous and abundant cytosolic proteins of 12–15 kDa, found in all eukaryotic cells [1]–[3] and virus [4]. They are key regulators of actin cytoskeleton dynamics through their interaction to monomeric actin (G actin), and to a plethora of actin-binding proteins, which involve poly-L-proline (PLP) stretches [5]. Moreover, interaction with phosphatidyl inositol-4,5-bisphosphate, a major component of cell-signaling transduction pathways, is essential for the integration of stress responses through cytoskeleton rearrangement, in addition to processes such as cell movement and cytokinesis driven by actin polymerization dynamics [1].

Profilins regulate the pools of G actin able to recharge newly depolymerized ADP-actin monomers with ATP and driving their assembly onto existing free barbed ends. This function could be developed by several mechanisms, including simple monomer sequestration, catalytic enhancement of actin-bound adenine nucleotide exchange, and the coupling of monomer addition to the growing filament with ATP hydrolysis [6]. However, this mechanism is likely to vary between species and, perhaps, under different physiological conditions. In addition, profilin facilitates the activity of nucleators of actin polymerization [7].

Profilin sequences similarity among plants and phylogenetically unrelated sources (lower eukaryotes, plants and animals) is low, whereas that from higher plants may reach 50% or even higher [8]. Up until now, the crystallographic structure of only three plant profilins have been published, including those from *Arabidopsis thaliana* pollen – Ara t 8 allergen (PDB code 3nul), *Betula pendula* pollen – Bet v 2 allergen (1cqa), and *Hevea brasiliensis* latex – Hev b 8 allergen (1g5u) (www.pdb.org). Despite the low sequence similarity, the overall 3D-structure (fold) of these three profilins is similar. Profilins fold get into a compact globular structure consisting of a central seven-stranded antiparallel β-sheet enclosed by the N- and C-terminal α-helices on one side and one or two helices on the other side [9]. Plant profilins have been characterized by a specific binding pocket located near the actin-binding surface, which is not present in profilins from other organisms [10].

Pollen from wind-pollinated seed plants constitutes one of the most important elicitors of type I allergy worldwide [11]. The allergenic properties of pollen are not part of its biological function, but different proteins have been associated with allergy [12]. Profilin was first recognized as an allergen (called Bet v 2) in birch pollen [13], and later described as allergen in plant foods and latex [14]. This family of proteins has been reported as the third most prevalent plant food allergen, behind the prolamin and the Bet v 1 families [15]. Despite this fact, plant profilins are considered minor allergens, recognized by the IgEs from 10% to 20% of pollen-allergic patients. They have been named in correlative order, as they were identified in particular plant species, i.e. Ole e 2 for olive pollen profilin, Ara t 8 for *Arabidopsis thaliana* and Bet v 2 for *Betula pendula*.

Profilin are widely cross-reactive allergens not only among botanically unrelated pollen, but also between pollen and food, as well as between pollen and latex [16]. This cross-reactivity is correlated to the conservation of profilin sequences fragments, in addition to the similarity of the overall fold and the conservation of surface patches between plants and mammals, fungi, and amoeba profilins. However, IgEs against plant profilins are able to weakly bind to the human homolog [17]. Thus, no profilin from sources other than plants has been shown to elicit allergic reactions.

The wide spread cross-reactivity of profilins has led to the designation of profilins as ‘pan-allergens’ [18]. The sensitization to these allergens has been considered a risk factor for allergic reactions to multiple pollen and pollen-food associated sources, contributing to a major health problem [16].

In the present study, we have analyzed pollen profilin polymorphism and studied its influence over the structure of the profilin isoforms, the changes in ligand-interacting surfaces, and how both factors might increase profilin functional variability. We have carried out an extensive analysis of the conformational and lineal B- and T-cell epitopes polymorphism, to unravel common shared and isoform-specific epitopes, providing a comprehensive understanding of the broad cross-reactivity and specific allergy reactions to profilin isoforms. The knowledge provided in this study will help developing rational strategies to improve the component-resolving diagnosis and immunotherapy of pollen allergy.

## Results

### Searching for Ole e 2 templates

After searching for proteins with known tertiary structure in the Protein Data Bank (PDB), the profilins from *Arabidopsis thaliana* pollen – Ara t 8 allergen (GenBank accession number AAB39480.1), *Betula pendula* pollen – Bet v 2 allergen (AAA16522.1), and *Hevea brasiliensis* latex – Hev b 8 allergen (AJ243325.1), showed the highest sequence identities for all profilin sequences analyzed, ranging from 73 to 93% (Table S1). The suitability of the selected model was evaluated by BioInfoBank Metaserver, which returned 3D-Jury score (J-score) ranging 0.57 to 0.88. We also used the Swiss-model server to identify the best possible template to build all profilin structures, finding high scores and very low E-values (ranging 3.39E^−63^ to 7.01E^−52^) for the 1g5uA, 1cqa and 3nul templates retrieved from the PDB database and used for homology modeling (Table S1).

### Structural assessment of the Ole e 2 built models

Different tools were used to assess the quality of the models built for this study:

a) *Procheck analysis*. The main chain conformations of the profilin models were located in the acceptable regions of the Ramachandran plot. A majority of residues (74.5–91.2%) were in the most favorable regions, whereas 8.8–21.7% of the residues were placed in the allowed regions, and 0.0–2.8% were in generally allowed regions. On the contrary, only 0.0–1.0% of the residues were present in the disallowed regions. The plot of ×1 versus ×2 torsion angles for each residue showed that most of the rotamers in profilin models were localized in low energy regions. All main-chain and side-chain parameters were in the better region. The goodness factor (G-factor) is essentially a log odds score based on the observed distribution of stereochemical parameters such as main chain bond angles, bond length and phi–psi torsion angles. The observed G-factor scores of the present model ranged between −0.35 to 0.27 for dihedral bonds, and 0.02–0.43 for covalent bonds (−0.15 to 0.33 overall). The G-factor predicts the quality of overall bond and angle distances, which should be above −0.50 for a reliable model [19]. The average value of the distribution of the main chain bond lengths (99.4%) and bond angles (96.7%) were well within these limits.

Furthermore, residues in favorable (60.6, 89.5, and 88.5%), allowed (35.6, 10.5, and 11.5%), generally allowed (2.9, 0, and 0%) and disallowed (1.0, 0, and 0%) regions were assessed for the models 1g5uA, 1cqa, and 3nul, respectively.

b) *ProSa analysis* returned Z-scores of pair, surface and combined energy for modeled profilin structures between −5.85 and −7.90. All the residues of profilin structures showed negative interaction energy and comparable to the one revealed by ProSA web energy plots, within the lowest energy range. In addition, the Z-scores were within the range usually found for native proteins of similar size, i.e. −7.16, −5.50, and −7.33 for the models 1g5uA, 1cqa, and 3nul, respectively.

c) *QMEAN analysis*. Q values for profilin structures ranged between 0.628 and 0.815. Quality factors of 0.656, 0.789, and 0.787 were estimated for the crystal structures of the templates 1g5uA, 1cqa, and 3nul, respectively.

d) Root mean square deviations (RMSD) between the different profilin built structures and the crystal templates Cα backbones ranged 0.275–0.062 Å for 1g5uA, 0.545–0.145 Å for 1cqa, and 0.432–0.090 Å for 3nul.

### Polymorphism effects in the profilin structural elements

**Figure 1:**
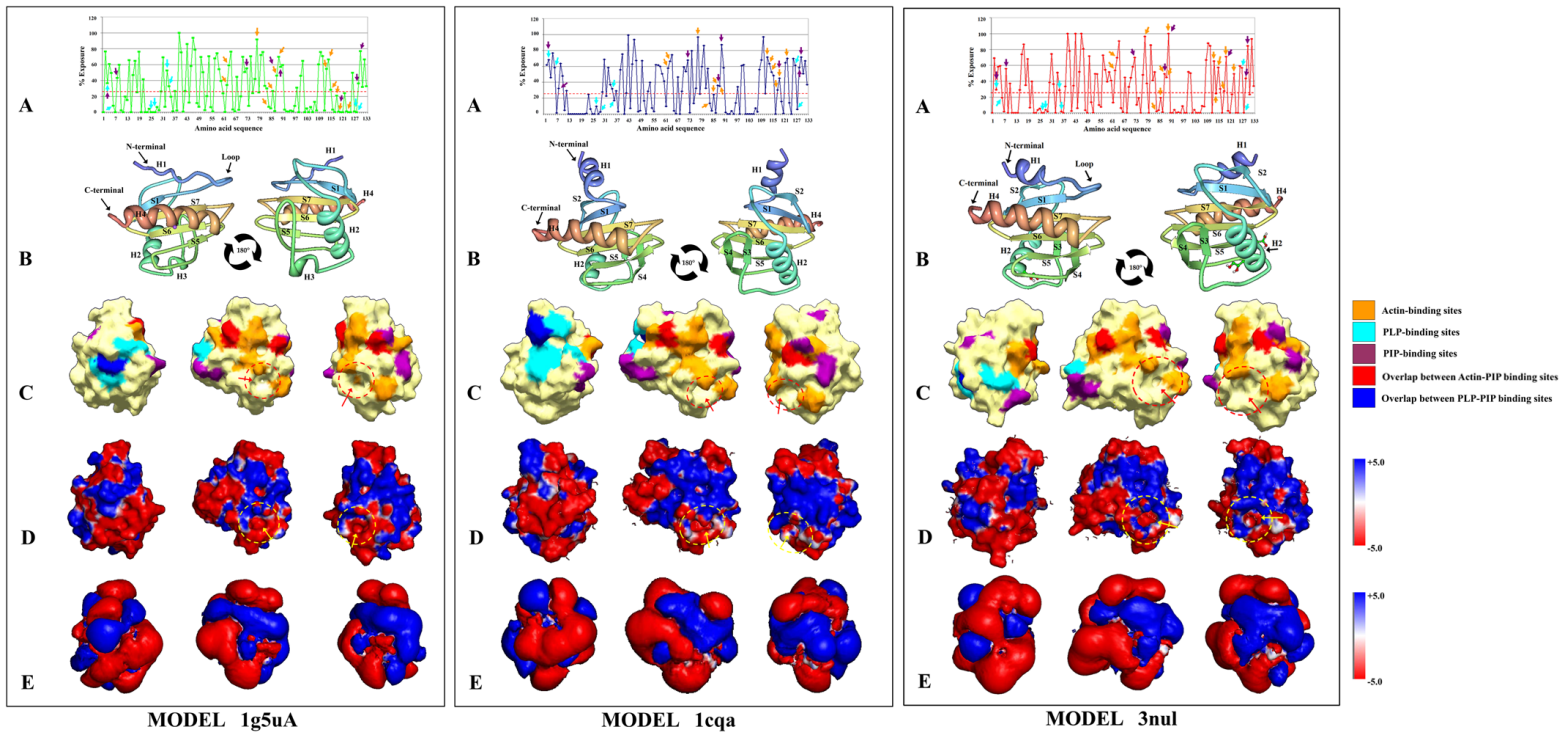
Surface distribution analysis of the profilin polymorphism. Different colors were used to highlight the different degree of variability over the surface for the three models used in this study, A) 1cqa, B) 1g5uA, and C) 3nul models. Residues which variability were high (variability index value, viv>3) were depicted in yellow color. Residues with intermediate (1<viv>3) and low (viv<1) variability were depicted as green and blue, respectively [102]–[104]. Surface residues implicated in ligand-binding domains (actin, PLP and/or PIP) were highlighted with transparent white shadows over the protein surface and discontinues borders lines. Red dotted circles and red arrows pointed a detailed plant specific solvent-filled cavity.

Protein models (Figure 1) were built by using crystal structure templates of plants profilins: (1) *Hevea brasiliensis* (Hev b 8.0204, PDB code 1g5uA), that shares a sequence identity of 74 to 88%, 86%, 76%, and 78 to 82% to Ole e 2, Cor a 2, Phl p 12 and Zea m 12 allergens, respectively; (2) *Betula pendula* pollen (Bet v 2, PDB code 1cqa), that shares a sequence identity of 80 to 83%, 92 to 93% and 88 to 90% to Ole e 2, Bet v 2 and Cor a 2 allergens, respectively; and (3) *Arabidopsis thaliana* pollen (Ara t 8, PDB code 3nul), that exhibits a sequence identity of 73 to 78%, 73% and 74 to 77% with sequences of Ole e 2, Cor a 2 and Phl p 12 allergens, respectively.

Despite the wide range of variability observed in the amino acid sequence [8], amino acids implicated in the maintenance of the general folding and the 3D-structure of profilins are well conserved. The analysis of the polymorphism affecting the structure resulted in a range of 1–3 variable residues in *Olea europaea* L., *Corylus avellana*, *Phleum pratense*, and *Zea mays* (Table S2). 20% of the sequences in *Corylus avellana* showed Ala in the position 25, 10% and 30% showed Gly in position 69 in *Phleum pratense* and *Zea mays*, respectively, in addition to 90% of the residues in position 72 in *Olea europaea* L.

Sequence polymorphism may also be responsible of the changes in the spatial distribution of the skeleton alpha carbons, which is reflected in differences between the structures of profilins. These differences can be measured by superimposition of structures using the RMSD parameter, which showed the following values: 3nul vs. 1cqa = 0.79 Å, 3nul vs. 1g5uA = 0.79 Å and 1g5uA vs. 1cqa = 0.87 Å.

Furthermore, one of the key forces in the maintenance of the proteins 3D-structure (the presence of intra-molecular disulphide bridges) was analyzed. Different number of cysteines was present in the sequences of different species (Table S3), among which the olive displayed the largest differences [8]. Depending on the olive cultivar analyzed, one to three cysteines were detected in 1.03%, 57.74% and 27.83% of the olive sequences, respectively. Further analysis of the possible combinations of SH-bonds indicated that the most feasible bond corresponding to that established between C^13^–C^118^ in profilin sequences containing 2 or 3 cysteines (Table S3A), which are the most energetically favorable.

The analysis of 2-D elements showed the most characteristic folding motifs of profilin, integrated by 7 β-strands sandwiched between the N- and C-terminal, nearly parallel N-terminal α-helices H1 and C-terminal H3 on one side and the middle perpendicular helix H2 on the other side [10]. Remarkable differences were found when individual sequences were analyzed. i.e. the N-terminal α-helix 1 was longer in those profilins of *Olea europaea* L. and *Betula pendula* built on the basis of the 1cqa model, in comparison to the other models (1g5uA and 3nul) (Table S1).

Our results indicate that polymorphism affected external loops of the profilins structure, particularly the loop comprised by the residues 18 to 20 (Figure 1). We found a deletion of one or three amino acids in those profilin sequences built on the basis of the 1cqa model in *Betulaceae* or *Poaceae* species and several other sequences of *Olea europaea* L. In addition, we found β-sheet 2 (residues 22 to 28) partially substituted by a α-helix in *Betula pendula* profilin sequences, and completely substituted in profilin sequences for *Phleum pratense* and *Corylus avellana* [8].

Moreover, olive profilin sequences which conserved the complete loop 1, exhibited micro-heterogeneities in this region. 70% of the profilin showed a motif ^18^HE**G** ^20^ and 14.5% showed the sequence ^18^HE**D** ^20^, changing a neutral residue as Gly (G) by and acidic residue such as Asp (D), with a voluminous radical. *Betula pendula* showed a deletion in this loop, losing the histidine in position 18, in addition to a micro-heterogeneity in position 20, ^19^Q**A** ^20^ and ^19^Q**G** ^20^. Two profilin sequences in *Corylus avellana* missed the three amino acids motif of this loop, and the rest of sequences showed a deletion in position 18, displaying the motif ^19^QG^20^.

The 3-D structure exhibited conspicuous differences (Figure 1). First, the number of exposed residues of profilin is different according to the templates used for modeling, making variable the contact surface of the profilin analyzed (Figure 1A). Second, there are noticeable differences in the spatial distribution and orientation of different 2-D elements such as the N-terminal α-helix, particularly in profilins built based in the 1cqa template. β-sheets 2, 3 and 4 are substituted by three loops in those profilins modeled on the basis of the 1g5uA model, and β-sheet 5 is shorter in these profilins compared to the ones built on the basis of the other two models (1cqa and 3nul). A small α-helix 3 is present in 1g5uA, but missing in profilins based in the 1cqa and 3nul models (Figure 1B).

**Figure 2:**
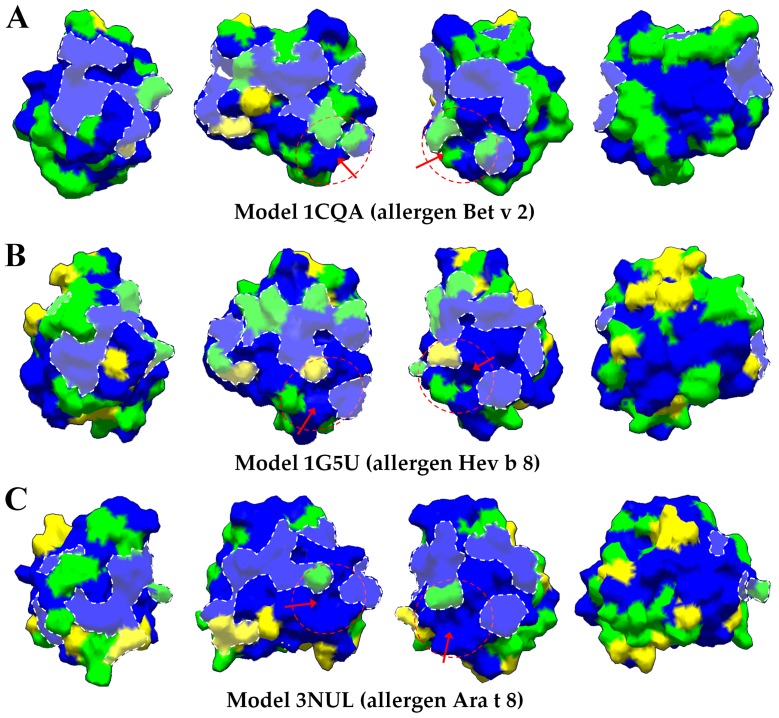
Profilin structure features, ligand-binding domains and electrostatic potential distribution. Left, central, and right panel represent to 1g5uA, 1cqa, and 3nul models, respectively. A) Solvent accessible surface area (SASA) calculated for the residues of each crystallographic model. Key amino acids implicated in Actin, PLP and PIP interaction are highlighted with orange, blue and purple arrows, respectively. A red dotted line delimited the residues with SASA>25%. B) Three-dimensional structure of profilin models 1g5uA, 1cqa, and 3nul (from left to right panel) showing two views rotated 180°. Different secondary structural elements such as α-helices, β-sheets, and loops are highlighted with letters H, S, respectively. All structures were depicted as a cartoon diagram. C) Surface representation views of the three models of profilins rotated 90°, showing the surface residues involved in the different ligand-binding surfaces such as actin (orange), PLP (light blue), and PIP (purple). Residues belonging to actin-PIP and PLP-PIP binding regions are highlighted with red and deep blue colors, respectively. Red dotted circles and red arrows point a detailed of the plant specific solvent-filled cavity. D) 90° rotated views of the electrostatic potential representation on the three profilin models surface, showing the plant specific solvent-filled cavity highlighted by yellow dotted lines and arrows. The surface colors are clamped at red (−5) or blue (+5). E) Electrostatic potential (isocontour value of ±5 kT/e) surface for the three models of profilins are depicted in 3 rotated 90° views.

A major structural difference between plant profilins and those profilins from other *Fila* is the presence of a plant specific solvent-filled pocket, previously identified in *Arabidopsis* [10]. This represents a unique feature of plant profilins, since it is missing from *Acanthamoeba* and bovine profilins [20], [21]. The conservational analysis of this signature showed that this is relatively well conserved among plant profilins. Only few variations were detected among the residues integrating the pocket, and they were particularly present in profilins built with the template 1g5uA (Figure 2).

### Polymorphism affecting ligands-binding surfaces: solvent accessible area, electrostatic potential and conservational analysis

A comparative analysis of the variability of profilin's key residues involved in the interaction with ligands such as actin, PLP and PIP (Figure 1) was depicted over the surface structure of the 1cqa (Figure 2A), 1g5uA (Figure 2B), and 3nul (Figure 2C), models. The degree of variability was highlighted with different colors, i.e. highly variable, viv>3 (yellow color), putative variable, 3<viv>2 (green color), and low variability, viv<1 (blue color). This analysis showed that most of the variable residues and putative variable residues (Figure 2) were present across the surface, and particularly near to the ligand-binding domains (Figure 1). Few of these variable residues were located within PIP-interacting areas, or in the regions of contact with actin (Figure 1B and Figure 2).

Furthermore, the analysis of the polymorphism present in the individual key amino acids of profilin with a central role in the interaction with actin (i.e. A^64^, P^65^, **Q** ^79^, V^85^, R^87^, K^89^, K^90^, T^114^, P^115^, G^116^, N^119^, M^120^, R^124^) [10], [22], proline-rich proteins (i.e. W^3^, Y^6^, I^28^, G^30^, W^36^, A^37^, Y^128^, L^129^) [10], [23], and phosphoinositide lipids (i.e. W^3^, D^8^, K^74^, K^89^, G^91^, M^120^, **L^130^**, **E^131^**) [24], [25], [26] showed that they also have a major contribution in terms of surface accessibility (SAS>25%) (Figure 1A: left, central and right panel). In particular, these residues displayed in bold presented a higher variability. Additionally, olive profilin sequences also exhibited high variability in the residues G^62^, Q^79^ and A^82^.

Our results indicated that the actin-binding surface was well conserved in all profilins. Only few variable residues, i.e. H^62^, Q^79^ were located in this area directly implicated in the interaction with actin and taking part of the plant specific solvent-filled pocket (Figure 1C; Figure 2). Analysis of the adjacent residues, which also integrate PLP- and PIP-binding regions, and maintain the connectivity and stability in these binding domains, revealed differences in the variability index, which may affect the interacting properties with natural partners [8].

The highest variability was found in the L^130^ and E^131^ residues located in the PIP-binding surface (Table S4A) for the 5 species analyzed, as well as some particular residues in olive profilins such as D^8^ and Q^79^ (Table S4B).

Furthermore, we analyzed the variability of the ^84^(A/V)VIRGKKG(T/S/A)GGIT(V/I)KKT^100^ motif, found in all plant profilin but PpPRO1 from *Phleum pratense* [27], which has been described to be involved in the phosphatidylinositol-4,5-bisphosphate functional interaction, and take part of the MAP kinase phosphorylation domain [28]. Micro-heterogeneities were found in two positions of the Phl p 12 profilins (A/V^84^→R, and I^86^→T, Uniprot accession number X77583 and DQ663541, respectively) from *Phleum pratense*, and five positions of the Ole e 2 profilin (I^86^→V, DQ317563; I^86^→T, DQ138358 and DQ663555; V/S^97^→S, DQ317574; V/S^97^→T, DQ317570; and K^99^→E, DQ138352 and DQ138354) from *Olea europaea* L.

Electrostatic potential analysis of profilins revealed a net charge of −8 (12.78% negative and 6.77% positive) for the group of profilins built with the template 1cqa, −7 (13.85% negative and 8.46% positive) for 3nul and 6 (12.21% negative and 7.63% positive) for 1g5uA (Figure 1D: left, central and right panel). These electrostatic potentials are in agreement with the average percentage of negative and positive charges for olive sequence: 11.53% and 6.72% for 1cqa, 10.25% and 6.27% for 3nul, 11.98% and 6.77% for 1g5uA, respectively. Actin surface is dominated by a negative potential in the five species analyzed. Major differences were found in the PLP and PIP binding surfaces. The PLP-binding surface is dominated by positive PB electrostatic potential in profilin built with the template 1g5uA (Figure 1D: left panel), and negative charge distribution was found mainly in profilins built with the other two templates (Figure 1D: central and right panel). PIP-binding regions displayed no differences (Figure 1D), since positive and negative residues were equally distributed. Isocontour values of ±5 kT/e were depicted to highlight charge nature of the profilin ligand-binding regions (Figure 1E).

**Figure 3:**
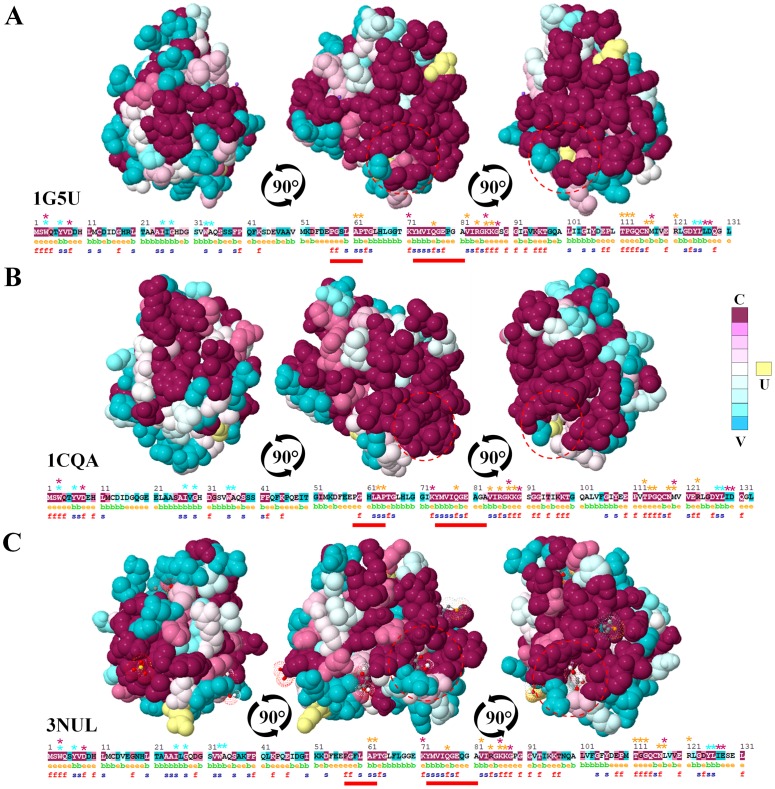
Phylogenetic analysis of olive profilin isoforms. Neighbor-joining (NJ) method was used to perform a phylogenetic analysis of the deduced protein sequences of *Olea europaea* L. profilin from 24 different cultivars. Each group of proteins are characterized by the 3D structural similarity represented by the PDB models A) 3nul of the Ara t 8 allergen, B) 1g5uA of Hev b 8 allergen, and C) 1cqa of Bet v 2 allergen. Profilin sequences from the same olive cultivar are highlighted with red arrows.

Consurf conservational analysis of structural and functional key amino acids showed that residues implicated in plant profilins fold maintenance were well conserved, but several variable residues were located in different superficial areas (Figure 3). As previously pointed out, few residues located in the plant specific binding pocket exhibited different degree of variability i.e. H^62^, Q^79^ (Figure 1 and Figure 3). In addition, particular olive cultivars such as ‘Bella de España’, ‘Sourani’, ‘Picual’ and ‘Lucio’, as well as maize profilin sequences showed highly variable residue in this area, i.e. H^62^ and P^82^ (Figure 1 and Figure 3).

### Phylogenetic analysis

**Figure 4:**
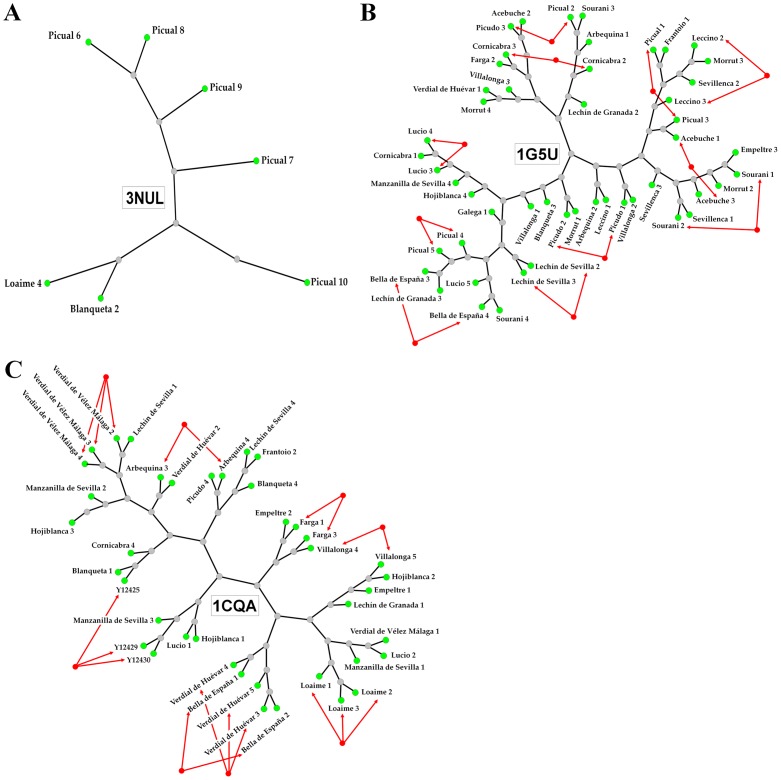
Profilin conservational analysis. Consurf-conservational analysis of profilin proteins showed in three individual views rotated 90° for the PDB models A) 1g5uA, B) 1cqa, and C) 3nul, respectively. The conserved and variable residues are presented as space-filled models and colored according to the conservation scores. The strictly conserved and variable residues are depicted in purple and blue, respectively. Red dotted circles and red arrows point a detailed of the plant specific solvent-filled. The sequence of the protein is depicted with the evolutionary rates color-coded onto each site. The residues of the query sequence are always numbered starting from 1. The predicted burial status of the site (i.e. “b”-buried vs. “e”-exposed) is annotated under the sequence. Residues predicted to be structurally and functionally important, “s” and “f”, are also pointed out under the sequence. Amino-acid sites categorized as “Insufficient data” are colored in yellow, indicating that the calculation for these sites were generated using only a few of the homologous sequences. Orange, light blue and purple starts highlight the key amino acids implicated in the interaction with actin, PLP and PIP, respectively. Red lines under the sequences represent the profilin characteristic motif, which define this family of proteins. **C** = conserved, **V** = variable, **U** = undefined.

To assess the relationships between olive cultivars, and to perform individual clustering analyses profilins sequences were grouped according to the 3D-structure template (1cqa, 1g5uA or 3nul) used to build their structure (Table S1). These analyses showed relationships between olive cultivar sequences, since different branches of the trees displayed groups of profilins with similar genetic origin (highlighted with red arrows), similar physico-chemical properties (Mw and Ip), as well as other comparable properties such as number and combination of posttranslational motifs (Figure 4). Figure 4A showed a closed association for sequences of the cultivar ‘Picual’. Figure 4B highlighted the association of the cultivars ‘Lechin de Sevilla’, ‘Leccino’ and ‘Sourani’ sequences, and Figure 4C showed the same association for cultivars such as ‘Verdial de Vélez-Málaga’, ‘Verdial de Huevar’, ‘Loaime’, ‘Bella de España’ or ‘Farga’.

### Identification of highly antigenic regions in plant profilins

Physicochemical parameters such as hydrophilicity, accessibility, exposed surface, and antigenic propensity of polypeptide chains have been used to identify continuous epitopes. In this study, antigenicity determinants were targeted by locating the positive peaks in hydrophilicity plots, and identifying the regions of maximum potential of antigenicity (Figure S1). Kyte-Doolitte scale [29] was used to search hydrophobic regions in the proteins (Figure S1, section 1). Welling antigenicity plot [30] was used to assign an antigenicity value defined as the log of the quotient between the percentage of antigenicity in a sample of known antigenic regions and in average proteins (Figure S1, section 2). Accessibility of residues (Figure S1, section 3), and 2-D structural elements (Figure S1, section 4) were also plotted and used for the continue epitopes assessment.

We identified up to 8 regions (A1, A2, A4 to A7, B1, and B2) in *Olea europaea* L. profilins with high potential of antigenicity (Figure S1A), 7 regions (A1 to A7) in *Betula pendula* (Figure S1B), 7 regions (A1 to A7) in *Corylus avellana* (Figure S1C), 8 regions (A1 to A7, B3) in *Phleum pratense* (Figure S1D), and 7 regions (A1 to A7) in *Zea mays* profilin sequences (Figure S1E). These regions with high antigenicity correlated well with the B- and T-cell and conformational epitopes identified and analyzed in the present study. The most variable species in terms of number of different epitope forms for each antigenicity region corresponds to *Olea europaea* L. 10 different variable forms in the regions A2, with percentages of frequency ranging 64.05% to 1.03% were found, while the lowest variable specie was *Betula pendula* with 2 different variable regions and equal percentage, A2 (50% each antigenic form) (Table 1).

**Table 1:** Summary of the high antigenicity areas of profilin proteins sequences.

Specie	A1	A2	A3	A4	A5	A6	A7	B1	B2	B3
*Olea europaea* L.	A1-0 (23.71%) TYVDDH	A2-0 (1.03%) ELEGNPGHHLSA	-	A4-0 (78.35%) DFNEPGHLAPTGLHLG	A5-0 (11.34%) IRGKKGA	A6-0 (92.78%) EPVTPG	A7-0 (94.85%) ERLGDY	B1-0 (97.94%) FKPEE	B2-0 (92.78%) IKKTG	-
	A1-1 (63.92%) AYVDDH	A2-1 (64.95%) DIEGHEGHRLTA	-	A4-1 (9.28%) DFDEPGHLAPTGMFVA	A5-1 (85.575%) IRGKKGS	A6-1 (3.09%) EPMTPG	A7-1 (2.06%) EGLGDY	B1-1 (1.03%) VKPEE	B2-1 (3.09%) VKKTG	-
	A1-2 (6.19%) AYVDEH	A2-2 (14.43%) DIEGHEDHRLTA	-	A4-2 (7.22%) DFDEPGSLAPTGLHLG	A5-2 (1.03%) VRGKKGA	A6-2 (2.06%) EPLTG	A7-2 (1.03%) KRLGDY	B1-2 (1.03%) FKPDE	B2-2 (2.06%) IKETG	-
	A1-3 (3.09%) TYVDEH	A2-3 (8.25%) DIEGHQLGSAAI	-	A4-3 (3.09%) DFSEPGHLAPTGLHLG	A5-3 (1.03%) TRGKKGS	A6-3 (1.03%) ESVTPG	A7-3 (1.03%) ERLEDY	-	B2-3 (1.03%) SKKTG	-
	A1-4 (1.03%) AYVYEH	A2-4 (2.06%) DIEGQHLTAAAI	-	A4-4 (1.03%) DSNEPGHLAPTGLHLG	A5-4 (1.03%) VRGKKGS	A6-4 (1.03%) EPVAPG	A7-4 (1.03%) GRLGDY	-	B2-4 (1.03%) TKKTG	-
	A1-5 (1.03%) GYVDDH	A2-5 (1.03%) DIEGPEDHRLTA	-	A4-5 (1.03%) DFNEPGHLAPTGLHLGG	-	-	-	-	-	-
	A1-6 (1.03%) SYVDDH	A2-6 (1.03%) EIESHHLSSAAI	-	-	-	-	-	-	-	-
	-	A2-7 (1.03%) DIEDHEGHRLTA	-	-	-	-	-	-	-	-
	-	A2-8 (1.03%) EIEGLHLASTAI	-	-	-	-	-	-	-	-
	-	A2-9 (1.03%) DLEGNPGHHLAA	-	-	-	-	-	-	-	-
	-	A2-10 (1.03%) DIEGQHLTAAAV	-	-	-	-	-	-	-	-
*Betula pendula*	A1-0 (100%) TYVDEHL	A2-0(50%) DGQASNSLA	A3-0 (100%) DGSVWAQSSSF	A4-0 (100%) EPGHLAPTGLHL	A5-0 (100%) VIRGKK	A6-0 (100%) KKTGQ	A7-0 (100%) ERLGDY	-	-	-
	-	A2-1 (50%) DGQGQQLAA	-	-	-	-	-	-	-	-
*Corylus avellana*	A1-0 (70%) AYVDEHL	A2-0 (80%) GQQLAAS	A3-0 (80%) DGSVWAQSSSF	A4-0 (80%) EPGHLAPTGLHL	A5-0 (90%) AVIRGKKG	A6-0 (90%) IKKTGQ	A7-0 (90%) ERLGDY	-	-	-
	A1-1 (30%) TYVDEHL	A2-1 (10%) GHHLSAA	A3-1 (10%) DGSVWAQSSTF	A4-1 (10%) EPGSLAPTGLHL	A5-1 (10%) VVIRGKKG	A6-1 (10%) VKKTSQ	A7-1 (10%) VGRLGDY	-	-	-
	-	A2-2 (10%) GHHLASA	A3-2 (10%) DGTVWAQSADF	A4-2 (10%) EPGHLAPTGMFV	-	-	-	-	-	-
*Phleum pratense*	A1-0 (60%) TYVDEH	A2-0 (100%) EGHHLAS	A3-0 (100%) DGTVWAQSAD	A4-0 (91.67%) KDFDEPGHL	A5-0 (83.32%) AVIRGKKGA	A6-0 (100%) IKKTGQ	A7-0 (100%) VERLGDYL	-	-	B3-0 (41.67%) VAAAKY
	A1-1 (60%) AYVDEH	-	-	A4-1 (8.33%) KDLDEPGHL	A5-1 (8.34%) RVIRGKKGA	-	-	-	-	B3-1 (33.33%) VATAKY
		-	-	-	A5-2 (8.34%) AVTRGKKGA	-	-	-	-	B3-2 (25%) VAGAKY
*Zea mays*	A1-0 (70%) AYVDEHL	A2-0 (70%) EGHHLTS	A3-0 (90%) GHDGAAWAQS	A4-0 (30%) DEPGHLA	A5-0 (100%) AVIRGKKGS	A6-0 (100%) ITVKKTGQ	A7-0 (100%) VERLGDYL		-	-
	A1-1 (30%) TYVDEHL	A2-1 (20%) EGHHLSS	A3-1 (10%) GHDGATWAQS	A4-1 (70%) DEPGFLA	-	-	-	-	-	-
	-	A2-2 (10%) EGHHLAA	-	-	-	-	-	-	-	-

The relative frequency of each isoform was calculated in percentage, and they have been distinguished with a suffix (0-10), where 0 represent the form designed as template for each specie and area.

### Analysis of B-cell epitopes

**Figure 5:**
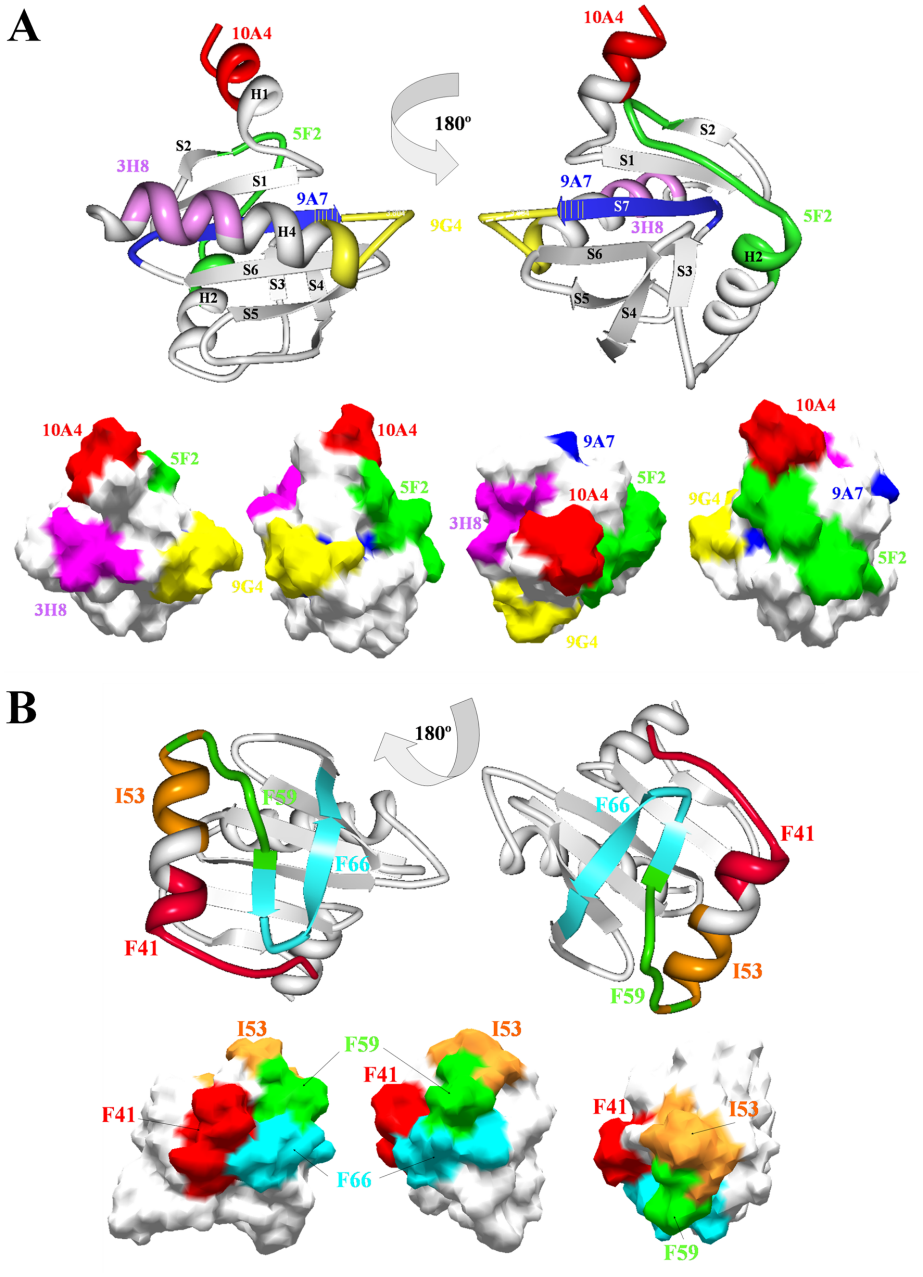
B- and T-cell epitopes superimposition on the surface of the profilin structures. A) Cartoon representation of profilin model 1cqa two views rotated 180° respectively, showing the localization of 5 B-cell epitopes, 10A4 (red), 5F2 (green), 9A7 (blue), 9G4 (yellow), and 3H8 (pink), in the 2-D structural elements of the protein. Overlapping sequence of 9A7 and 9G4 epitopes are depicted with vertical yellow lines. All epitopes are integrated by final part of two α-helices and its corresponding flanking loops, or a β-sheet. Surface superimposition of epitopes shows a broad distribution. B) Cartoon representation of profilin model 1cqa two views rotated 180° respectively, showing the localization of 5 T-cell specie-specific epitopes, I^53^ (orange) by *Olea europaea* L., F^41^ (red) for *Betula pendula*, F^66^ (light blue) for *Phleum pratense*, and F^59^ (green), for *Zea mays*, in the 2-D structural elements of the protein. Partial overlapping epitopes are I^53^ and F^56^. Surface superimposition of epitopes shows the distribution in a specific area of the protein and not overlapping with B-cell epitopes.

Seven antigenic regions, 10A4 (63–73), 5F2 (85–100), 9A7 (100–110), 9G4 (117–129), and 3H8 (163–175) prone to B-cell binding were analyzed in the five species (Table 2). B-cell epitopes were superimposed over the surface of the protein model 1cqa (Figure 5A) to compare their surface distribution with T-cell (Figure 5B) and conformational (Table 3) epitopes.

**Table 2:** Summary of the variability in B-cell epitopes of profilin proteins sequences.

Specie	B-cell epitopes				
	10A4 MSWQAYV	5F2 AQSAKFPQFKPEEM	9A7 GQAMIMGIYD	9G4 YDEPVAPG	3H8 ERLGDY
***Olea europaea*** ** L.**	10A4-0 (67.02%) MSWQAYV	-	-	-	3H8-0 (96.91%) ERLGDY
	10A4-1 (25.77%) MSWQ**T**YV	5F2-1 (82.50%) AQSA**T**FPQFKPEEM	9A7-1 (56.74%) GQA**LVF**GIY**E**	9G4-1 (82.48%) Y**E**EPV**T**PG	3H8-1 (1.03%) E**G**LGDY
	10A4-2 (4.12%) M**L**WQAYV	5F2-2 (5.14%) AQSA**D**FPQFKPEE**I**	9A7-2 (26.80%) GQA**LVC**GIY**E**	9G4-2 (10.31%) YDEP**MT**PG	3H8-2 (1.03%) ERL**E**DY
	10A4-3 (1.03%) MSW**H**AYV	5F2-3 (3.09%) AQSA**T**FPQFKPEE**V**	9A7-3 (9.28%) GQA**LVV**GIYD	9G4-3 (2.06%) YDEP**LT**PG	3H8-3 (1.03%) **K**RLGDY
	10A4-4 (1.03%) MSWQ**G**YV	5F2-4 (3.09%) AQSA**TA**PQFKPEE**I**	9A7-4 (3.06%) GQA**LIF**GIYD	9G4-4 (4.12%) Y**K**EPV**T**PG	-
	10A4-5 (1.03%) MSW**PT**YV	5F2-5 (1.03%) AQSA**TS**PQFKPEEM	9A7-5 (2.06%) GQA**LVC**GIY**K**	9G4-5 (1.03%) Y**E**EP**LT**PG	-
	-	5F2-6 (1.03%) AQS**TA**FPQFKPEEM	9A7-6 (1.03%) GQA**LVF**GIY**K**	-	-
	-	5F2-7 (1.03%) AQSA**T**FPQFKP**V**EM	9A7-7 (1.03%) GQAM**VV**GIYD	-	-
	-	5F2-8 (1.03%) AQSA**T**FPQFKP**D**E**I**	-	-	-
	-	5F2-9 (1.03%) AQS**SA**FPQFKPEEM	-	-	-
	-	5F2-10 (1.03%) AQS**TA**FPQFK**T**EE**I**	-	-	-
***Betula pendula***	10A4-0 (100%) MSWQ**T**YV	5F2-0 (100%) AQS**SS**FPQFKP**Q**E**I**	9A7-0 (100%) GQA**LVF**GIY**E**	9G4-0 (100%) Y**E**EPV**T**PG	3H8-0 (100%) ERLGDY
***Corylus avellana***	10A4-0 (60%) MSWQAYV	-			3H8-0 (100%) ERLGDY
	10A4-1 (40%) MSWQ**T**YV	5F2-1 (80%) AQS**SS**FPQ**L**KPEE**I**	9A7-1 (80%) GQA**LVF**GIY**E**	9G4-1 (80%) Y**E**EPV**T**PG	-
	-	5F2-2 (10%) AQS**ST**FPQFKPEE**I**	9A7-2 (10%) **S**QA**LIF**GIYD	9G4-2 (10%) YDEP**LT**PG	-
	-	5F2-3 (10%) AQSA**D**FPQFKPEE**I**	9A7-3 (10%) GQA**LVV**GIYD	9G4-3 (10%) YDEP**MT**PG	-
***Phleum pratense***	10A4-0 (58.33%) MSWQAYV	-	-	-	3H8-0 (100%) ERLGDY
	10A4-1 (41.67%) MSWQ**T**YV	5F2-1 (100%) A**DFPQ**F**KPEEITGI**	9A7-1 (100%) GQA**LVV**GIYD	9G4-1 (100%) YDEP**MT**PG	-
***Zea mays***	10A4-0 (70%) MSWQAYV	-	-	-	3H8-0 (100%) ERLGDY
	10A4-1 (30%) MSWQ**T**YV	5F2-1 (40%) AQS**TA**FPQFK**T**EEM	9A7-1 (40%) GQA**LVI**GIYD	9G4-1 (100%) YDEP**MT**PG	-
	-	5F2-2 (20%) AQS**TA**FP**E**FK**T**E**D**M	9A7-2 (30%) GQAM**VV**GIYD	-	-
	-	5F2-3 (20%) AQS**TA**FPQFK**P**EEM	9A7-3 (20%) GQA**LVV**GIYD	-	-
	-	5F2-4 (10%) AQS**TA**FP**E**FKPEEM	9A7-4 (10%) GQA**LII**GIY**S**	-	-
	-	5F2-5 (10%) AQS**TA**FPQ**S**K**T**EEM	-	-	-

The relative frequency of each isoform was calculated in percentage, and they have been distinguished with a suffix (0-10). Variable residues have been highlighted with bold and bigger size letters.

**Table 3:** Conformational epitopes of profilin proteins sequences.

*Hevea brasiliensis* (Hev b 8 allergen)			
Epitope	Central residue	Conformational epitopes sequences	N° of residues
1	S2	**_2_S**WQTYVDDH_10_ Q_35_ Y_125__129_QGL_131_	14
2	R19	_8_DDH_10__13_CDIDGHRLT_21__37_SS_38__107_DEPLT_111_	19
3	S37+S38	Q_4_ D_8__19_RLT_21_ V_32__35_QSSSFPQ_41_ G_69_ D_107_	15
4	Q41	_30_GSV_32__35_QSSSFPQFKSD_45_ H_66__69_GT_70_	17
5	S44+D45	_28_HDGSV_32__40_PQFK**S**D**_45_**_48_AAVMK_52_ T**_63_** H_66_ G_69_	19
6	T63	D_45__48_AA_49_ K_52__58_GSLAPT_63_ H_66__69_GTKYMV_74_	17
7	Q76	_57_PG_58_ A_61__73_MVIQGEPGA_81__84_RGK_86_ P_112_ N_116_ E_120_	18
8	P79	_51_MK_52__55_DEPGS_59__74_VIQGEPGA_81_ R_84_ K_96_ E_120_	18
9	E108	_17_GHRLT_21__86_KKGS_89__107_DEPLTP_112_	15
10	M117	H_10__13_CDIDGH_18_ R_84__111_TPG_113__116_NM**_117_**_120_ER_121__124_DY_125_	17
11	D128	Y_6__96_KTGQ_99__120_ER_121__124_DYLLDQGL_131_	15

*Betula pendula* (Bet v 2 allergen)			
Epitope	Central residue	Conformational epitopes sequences	N° of residues
1	T5	_2_SWQTYVDEHLM_12__37_QSS_39_	14
2	S40	Q_4__8_DELM_12_ A_23__37_QSSSFPQ_43_ I_72_ K_89_ E_109_	16
3	Q43	_32_GSV_34__37_QSSSFPQFKPQ_47__68_HLGGI_72_	19
4	P46	_30_HDGSV_34__42_PQFKPQ_47_ T_50__53_MK_54__68_HLGG_71_	19
5	M75	_63_APT_65_ H_68__72_IKYMVIQ_78__86_RGKKG_90_	16
6	Q78	A_63__75_MVIQGE_80__82_GAVIRGK_88_ P_114_ N_118_	16
7	A81	_53_MK_54__57_EEEGH_61__77_IQGEAGA_83_ R_86_ K_98_	16
8	G90	_72_IKYM_75_ I_77__88_KKGSG_92__109_EE_110_ V_112_ P_114_	13
9	P111	A_23_ S_91__109_EEPVTPGE_116_	10

*Arabidopsis thaliana* (Ara t 8 allergen)			
Epitope	Central residue	Conformational epitopes sequences	N° of residues
1	G17	_15_VEGNH_19__107_DEPMT_111_ Q_114_	11
2	A37+K38	Q_4_ D_8_ H_19_ T_21_ V_32__36_SAKFPQ_41_ G_68_ E_70_ D_107_	14
3	Q41	G_30_ V_32__36_SAKFPQLKPQ_45__66_FLGGE_70_	17
4	P44+Q45	_28_QDG_30__40_PQ_41__43_KPQ**_45_** D_48__51_KK_52_ F_59__66_FLGGE_70_	17
5	T63	_58_GFLAPT**_63_** F_66__69_GEK I_75_ K_86_	12
6	Q76	_57_PG_58_ A_61__75_IQGEQ_79_ K_86__112_GG_113_ N_116_	12
7	Q79	_51_KK_52__55_EEKPGF_59__75_IQG_77__79_QGA_81_	14
8	G88	_70_EK_71_ I_75__86_KK_87_ G_88__107_DE_108__111_TG_112_	10
9	E108+P109	_14_VEGNHLT_21__86_KKG_88__107_DEPMTGGQ_114_	18
10	E128+E130	W_3_ Q_28_ W_33__120_ER_121__124_DYLIESEL_131__98_NQ_99_	15

Central residue of each epitope is highlighted with a box. Epitopes were built in the allergenic proteins Hev b 8 (*Hevea brasiliensis*, AJ243325, Q9LEI8), Bet v 2 (*Betula pendula*, M65179, P25816), and Ara t 8 (*Arabidopsis thaliana*, U43325, Q42449).

Polymorphism analysis of B-cell epitopes in olive profilin showed low variability for 3H8 epitope. Large number of changes was detected for 10A4, 5F2 and 9G4 epitopes, being 9A7 the most variable region among all species analyzed (Table 2).

The highest variability in the number of different epitope forms for each B-cell epitope corresponded to *Olea europaea* L. profilins, particularly 5F2, with 10 different variable regions, and percentages of frequency among sequences ranging 82.50% to 1.03%. From these, 5F2-1 and 5F2-10 represented the higher and lower widespread variants, respectively. The 9A7 epitope was the second region with the higher number of epitope forms (precisely 7), with percentages ranging 56.74% (9A7-1) to 1.03% (9A7-6 and 9A7-7). The species with the lowest variability was *Betula pendula*, with only one region for each B-cell epitope (Table 2).

### Identification of T-cell epitopes

Variable number of anchor motifs to HLA-DR (T-cell epitopes) was found in the sequences of profilins of the five species analyzed (Table 4). T-cell epitopes were superimposed over the surface of the protein model 1cqa (Figure 5B) to compare their distribution compared to that of B-cell (Figure 5A) and conformational (Table 3) epitopes. T-cell epitopes were located in a delimited area of profilin, with few residues overlapping with B-cell epitopes.

**Table 4:** Comparison of anchor motifs to HLA-DR class II (T-cell epitopes) in the sequences of profilin proteins sequences.

A)				
*Olea europaea* L.	*Betula pendula*	*Corylus avellana*	*Phleum pratense*	*Zea mays*
V29	V28	V28	-	V26
-	W35	W35	-	-
-	F41	-	-	-
F45	F44	-	F42	F39
I53	-	-	-	-
-	-	-	-	F59
L68	-	-	M65	L65
-	-	-	F66	-
L70	**L69**	**L69**	**V67**	**L67**
M76	**M75**	**M75**	**M73**	**M73**
I86	-	-	I83	I83
I95	I94	I94	I92	-
				

B)									
*Olea europaea* L. Cultivars	First residue of the HLD-DR class II anchor motifs								
	V29	F45	I53	L68	L70	M76	I86	I95	I106
‘Acebuche’									X
‘Arbequina’									X
‘Bella de España’			X						
‘Blanqueta’			X						
‘Lechin de Granada’							X		
‘Lechin de Sevilla’							X		
‘Morrut’									X
‘Picual’	X		X						X
‘Picudo’									X
‘Sevillenca’									X
‘Sourani’									X
‘Verdial de Velez-Malaga’							X		

A) Initial amino acid of the anchor motifs to HLA-DR class II with high frequency (frequency>70%) among profilin sequences. Specific motifs are highlighted with boxes, and these common shared among all species are highlighted in bold. B) Comparison of anchor motifs to HLA-DR class II more frequent in the sequences of profilins for different olive cultivars. The initial residue of the anchor motifs for specific and common shared T-cell epitopes, which frequency in lower than 30% among profilin sequences of different cultivars are indicated with X.

Most profilin sequences analyzed contained commonly shared anchor motifs of HLA-DR class II (>70%) present in all species (L^70^ and M^76^), while others motifs were species-specific, such as F^41^ for *Betula pendula*, I^53^ for *Olea europaea* L., F^59^ for *Zea mays* or F^66^ for *Phleum pratense*. *Corylus avellana* was the only species without specific motifs, although W^35^ was only shared by the *Betulaceae* species (Table 3, Hev b 8 allergen conformational epitopes).

**Figure 6:**
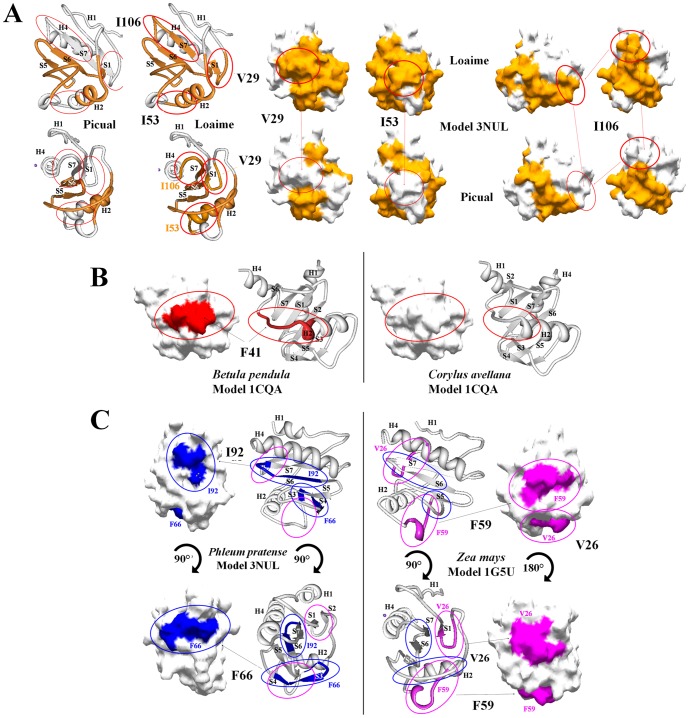
Olive cultivars and species specific distribution of T-cell epitopes. A) Cartoon representation of profilin model 3nul two views rotated 45° respectively two examples of olive cultivars, ‘Picual’ and ‘Loaime’, to compare the localization in the 2-D structural elements of the protein of the common shared T-cell epitopes between both cultivars, and the specific epitopes (V^29^, I^53^ and I^106^) only present in Loaime cultivar. All epitopes were depicted in orange color. Surface superimposition of both, common and not shared epitopes, are depicted in the same color over the model 3nul of profilin. Red circles were used to highlight the specific epitopes. B) Cartoon representation of profilin model 1cqa of the same view for both species of the *Betulaceae* genus, *Betula pendula* and *Corylus avellana*, showing the specific T-cell epitope F^41^, only present in *Betula pendula*. Presence or absence of the F^41^ epitope was located and highlighted in the 2-D structural elements of the protein, as well as over the surface of the model by using red color and red circles. C) Specific epitopes location and comparison between two species of the genus *Poaceae*, *Phleum pratense* and *Zea mays*, by using cartoon representation of 2-D profilin elements or protein surface over the models 3nul and 1g5uA two views rotated 90° or 180°, respectively. Blue color over the model surface and blue circles were used to highlight *Phleum pratense* specific T-cell epitopes F^66^ and I^92^. Pink circles were used to highlight the absence of *Zea mays* specific T-cell epitopes V^26^ and F^59^ over the 3nul model. Reciprocity of colors was used to show the presence or absence of specific epitopes in the model 1g5uA for *Zea mays*.

The polymorphism analysis of T-cell epitopes among profilins sequences of different olive cultivars showed a high number of combinations for several anchor motifs either present in large (>70%) and low (<30%) number of sequences. For example, I^106^ were identified in cultivars such as ‘Bella de España’, ‘Picual’, ‘Arbequina’, ‘Lechin’, etc. (Table 3, Bet v 2 allergen conformational epitopes). On the other hand, V^29^ was found specifically in the cultivar ‘Picual’. These findings highlight the existence of clear differences in the number and type of epitopes with high and low representation among olive cultivars, as well as the different combination of epitopes among the population of profilin sequences. The Figure 6A depicts the location of present and absent T-cell epitopes in profilin of different olive cultivars. ‘Loaime’ V^29^, I^106^, and I^53^ are missing in the profilin sequences of the ‘Picual’ cultivar. These differences are also extensive to the *Betulaceae* family, where the F^41^ epitope was present in *Betula pendula*, and absent in *Corylus avellana* (Figure 6B). The *Poaceae* family also showed the F^66^ and I^92^ epitopes being present in *Phleum pratense* and absent in *Zea mays*, and the opposite situation for the T-cell epitopes V^26^ an F^59^ (Figure 6C).

### Identification and analysis of conformational profilin epitopes

Profilin conformational epitopes exhibited a wide distribution over the surface of profilins (Table 3). Several of these conformational epitopes overlapped, partially or totally with the sequence of linear B- or T-cells epitopes described in previous sections.

Up to 11 conformational epitopes were found for profilins built with the structural template of the Hev b 8 allergen, 9 epitopes for Bet v 2 allergen, and 10 epitopes for Ara t 8 allergen (Table 3), with a number of amino acids ranging from 14 to 19, 10 to 19, and 10 to 18, respectively for these templates. As example of polymorphism, conformational epitope 1 of profilins built with the 3nul template (Figure S2) integrates part of the plant characteristic loop situated between the N-terminal α-helix 1 and β-strand 1, which frequently exhibited insertions of three to six amino acids in plant profilins, an numerous micro-heterogeneities in the residues 18 to 21, as previously described in the current study.

A detailed comparative analysis of conformational and linear epitopes recognized by B- and T-cell (Table S5) showed a high degree of overlap. Frequently, both types of linear B- and T-cell epitopes were partially overlapping in the same conformational epitope. In addition, conformational epitopes that overlapped only with B-cell epitopes, (i.e. C1), or T-cell epitopes, (i.e. C8), both in profilins of *Olea europaea* L. built with the Hev b 8 allergen model, were depicted in the Table 5.

**Table 5:** Conformational and lineal epitopes relationships.

A)					
Profilin model	Conformational and B-cell epitopes overlapping				
	*Olea europaea* L.	*Betula pendula*	*Corylus avellana*	*Phleum pratense*	*Zea mays*
Hev b 8	1 and 2	-	1	1, 2, 3 and 10	1, 2, 10 and 11
Bet v 2	1	1	-	-	-
Ara t 8	-	-	10	-	-

B)					
Profilin model	Conformational and T-cell epitopes overlapping				
	*Olea europaea* L.	*Betula pendula*	*Corylus avellana*	*Phleum pratense*	*Zea mays*
Hev b 8	8	-	7 and 8	5, 7, 8 and 11	7 and 8
Bet v 2	5 and 7	5, 6 and 7	6 and 7	-	-
Ara t 8	5 and 7	-	5 and 7	5, 6 and 7	-

A) Conformational epitopes overlapping totally or partially with lineal B-cell epitopes. B) Conformational epitopes total or partially overlapping with lineal T-cell epitopes.

Finally, we also found three conformational epitopes (epitope 1 from the model 3nul) in *Olea europaea* L., *Phleum pratense* and *Corylus avellana*, which did not overlap with either B- or T-cell linear epitopes. The central residue of this epitope 1, G^17^, belongs to the plant profilin characteristic loop situated between the position 18 and 21 (Figure S2), which exhibited high variability in the number and type of residues.

## Discussion

### Polymorphism affecting ligand-binding domains and structural features might contribute to generate functional variability among profilin

The identification of profilin sequences in databases has focused on data comparison searches for sequence homology within the open reading frames of profilin genes. Profilin sequences from diverse origins (not only from species distantly related) may show less than 25% overall homology [31]. Profilins within a given kingdom display higher sequence homology than those between different ones. This is particularly evident when the comparison is made between plant and animal profilins, particularly for the actin-binding surface of plant profilins, which is only partially conserved in animal profilins [10].

Profilin from mammals and plants exhibit a well conserved overall fold. However, major differences have been observed in structural 2-D elements, particularly when their length and spatial distribution are compared among different classes of profilin [9], [10], [32], [33]. Many of these differences are located within three characteristic loops, which allow distinguishing plant profilins from those of other kingdoms [10]. Two of these loops form the characteristic plant solvent-filled pocket, identified for the first time in *Arabidopsis* [10]. This pocket integrates part of the actin-binding domain. Our results indicated that the first loop between β-sheet 4 and 5 showed a high level of polymorphism in the surface residues. The level of variability was higher compared with the second loop situated between β-strands 5 and 6, close the PIP binding domain. In addition, the third loop situated between the N-terminal α-helix 1 and β-strand 1, presented a high degree of variability [10], and a characteristic insertion of three to six amino acids. This particular insertion is missing in several olive profilin sequences [8], [34], [35]. Based on the wide distribution and frequency of micro-heterogeneities of plant profilin (i.e. tobacco [36], and olive [8], [34], [35]), the presence of both common but also differential functional features among the plethora of plant profilin isoforms could be expected. However functional redundancy may be conceivable among these isoforms, i.e. profilins from unrelated species (plants and mammals) which share low sequence identity (≈20%) can fulfill similar functions [37], in addition to substitute each other in living cells [38]. These observations are also supported by the structural similarities observed between *Acanthamoeba* and mammalian profilins [39], [40].

On the other hand, the analysis of profilin 3D structure and surface characteristics may point out and supports the existence of specific isoforms of profilins with differential functionality, due to specific location of micro-heterogeneities, particularly affecting the 2-D elements and structural interacting surface domains with natural ligands. Multiple sequences of olive profilin have been found to exhibit variations in the length of the 2-D elements and their spatial distribution, variable geometry of the surface-interacting domains and variation in the electrostatic potential. These 2-D elements are critical for the interaction with partners like actin or PLP [10]. In addition, they affect the activity/function of the protein and regulate these interactions. Local superimpositions of profilin structures were performed by the Cα of the amino acid sequences, which allowed distinguish several different differences affecting these 2-D structural elements. However, only small dissimilates in RMSD were observed, which confirmed the conservation of the general fold of the plant profilins. Furthermore, larger differences were observed in the profilin structures after performing superimpositions using the radical carbons of each amino acid, which led to major disparities in the contact surface of the interacting domains. Local differences (stereochemical properties) in 2-D structural elements have been also found in the N-terminal region (β-sheet 1), which is partially or totally substituted by and α-helix as consequence of the sequence variability. This structural substitution is in agreement with one amino acid deletion found in *Betula pendula* [8], [13], or three residues in *Corylus avellana* [8], *Phleum pratense* [8], [35], [41], and *Zea mays* [8], [42].

Little is known about the implications of the cysteines in the protein folding and 3D structure maintenance of profilin. Presence of hydrogen bonds and electrostatic interaction between different atoms are also common and important forces orchestrating protein structure [43]. Dimeric profilin forms of *Hevea brasiliensis* (crystal structure 1g5uA) showed 4 hydrogen bonds and 84 non-bonded contacts in between the α-helices H1 and H4, being these the major forces involved in maintaining the protein structure. This also occurs in other proline-rich proteins (http://www.cathdb.info/pdb/1g5u), while no disulphide bridges were found. Furthermore, human profilin II (hPROFII) contains three cysteine residues (C^12^, C^15^, y C^16^) located in a conserved loop in between α-helix H1 and β-sheet S1. No disulphide bridges were found in this structure, where loop conformation is preserved by a network of hydrogen bonds [44].

Olive profilins exhibit a variable number of cysteines among cultivars sequences. The most energetically favorable pair of cysteines to form a disulfide bridge involved the couple C^13^–C^118^ in the sequences containing 2 or 3 cysteines, which is also in agreement with previous observations [45]. Such a variable number of cysteines found among olive profilins would suggest that cysteines might have not a large relevance in profilin 3D structure and fold preservation. However, the presence of a disulphide bond would be necessary to maintain the correct distance between both the C- and N-terminal in order to preserve the stability of the PLP interacting surface [46]. The present study suggest that only a restricted number of disulfide bridges (Table S3) showed thermodynamical and stereological compatible values [47], particularly for the pair C^13^–C^118^ in olive, or C^13^ and C^115/1117^ in *Corylus avellana*, *Phleum pratense* and *Zea mays*.

Experimental data are still waiting for confirmation of profilin intra and/or intermolecular bonds. Up until now, some results have shed some light about the formation of multimeric structures in human [48], *B. pendula* and *A. vulgaris* [49] profilins. Hydrogen bonds and electrostatic interactions would play an important role in the formation of this macromolecular complex, since these forms are resistant to treatments with reducer agents [50]. However, arguments are in favor of the existence of cysteine bridges protected in cavities or clefts, safe from the effect of reducing agents. Thus, the formation of Cys - Cys bridges would be dependent on the cellular red-ox conditions of defined cellular compartments were they would be localized [51]. Moreover, the presence of dimeric and multimeric forms of profilin is not incompatible with a correct profilin functionality, since interaction with natural ligands still may occurs as previously found in human profilin I and II, which are able to induce actin polymerization [52]. Moreover, it has been demonstrated that tetrameric forms of human profilins are also implicated in the maintenance of cellular morphology and contribute to signaling pathways [53].

In the present study, the analysis of the polymorphism present in the olive profilin isoforms, and more precisely, in those motifs interacting with profilin natural-partners has revealed a different degree of variability in key residues involved in these interactions. The extension of this variability also affected to amino acids localized in close proximity to these interacting areas, with strong energetic and stereochemical influence in the structural maintenance of these motifs, although not directly implicated in the interaction surface itself. This variability would affect a number of interacting properties such as the affinity between profilin and different ligands (i.e. actin, ATP, PIP [42], [54]), generating differences among isoforms, and therefore contributing to increase the functional variability of profilins. In this regard, functional variability would be a cellular mechanism able to face different stress and physiological conditions [55].

The analysis of the extension of the polymorphism between profilin isoforms has shown that the actin binding motif is not totally conserved, particularly these residues located in the plant specific solvent-filled pocket [10], as well as those residues directly implicated in the regulation of profilin interactions with PIP [56] and PI3K [57]. On the contrary, residues involved in binding PLP-stretches have shown to be not particularly affected by polymorphism. Interactions of profilin isoforms with numerous proline-rich proteins are finely regulated by phosphorylation, which may change the affinity properties of these interactions [57], [58]. Clear examples of targeting residues susceptible of phosphorylation are tyrosines 6 and 128 in olive cultivars [8]. Changes in these two residues may indicate that polymorphism somehow is implicated the regulation of these interactions by generating posttranslational variability instead of a direct implication in the PLP-profilin interaction, since no residues directly implicated in this interaction were affected by polymorphism [59]. Thus, phosphorylation variability within PLP domains might be a fundamental regulatory process, able to generate additional, differential interacting properties [60], and regulate profilin activities under different environmental conditions with different partners [61].

Polymorphism analysis of phosphoinositides lipid interacting surface in profilin showed high variability, particularly for the residues Leu^130^ and Glu^131^, directly implicated in this interaction. The variability of these residues may be the final responsible for the regulation of this interaction [31], [52], [62], since mutagenesis in these positions have shown change affinity properties of different profilin isoforms for different PIP lipids species such as (PI_(3,4)_P_2_ and PI_(3,4,5)_P_2_) [63]. Thus, differences in the affinity for PIP molecular interactions would constitute a mechanism to regulate the cellular integration of signal transduction under different cellular stresses and physiological conditions.

### Polymorphism is responsible of the generation of multiple epitopes, which may involve both specific and wide cross-reactivity to profilin isoforms

Pan-allergens as profilin are the most broadly distributed cross-reactive allergens throughout the animal and plant kingdoms [64]. Profilins have been described in a wide variety of plant sources [13], [17], showing highly conserved and variable regions, features that may contribute to their wide cross-reactivity, as well as isoform specific reactions [8, current study].

High degree of polymorphism has been described in plant pollen allergens from different sources: grass, Poa p 9 [65]; ragweed, Amb a 1 [66]; and trees, Bet v 1 [67], Ole e 1 [68], Ole e 11 [69]. Moreover, four recombinant isoforms of Cor a 1 from hazel pollen displayed different antigenic and allergenic properties due to differential changes in few amino acids [70]. The origin of this polymorphism in olive has been demonstrated for different allergens, such as Ole e 1 [68] and Ole e 2 [8, current study], where the genetic background of *Olea europaea* L. is the major source of sequences variability. In several cases, allergen polymorphism has been attributed to the presence of multigene families [71]. In other allergens, the presence of post-translational modifications may also determine the presence of multiple isoforms of the allergen, i.e. Ole e 1 [68], Ole 2 [8], Ole e 11 [69], and also for apple (*Malus domestica*), where allelic diversity regarding this allergen (up to 18 Mal d 1 genes), has been considered as a major explanation for the considerable differences in allergenicity [72].

Profilin broad distribution has been confirmed among a large number of botanically unrelated plants, which may be another important factor responsible of an increasing IgE-mediated risk of multiple pollen sensitizations [73] and pollen-related food cross-reactions [74]. Nevertheless, diverse profilin-sensitized patients only may react to a small number of profilin-containing allergen sources, probably due to a concomitance with other pollinosis [75], or sensitization to specific epitopes [76].

B- and T-cell responses have a defining and differential recognition of antigenic epitopes, and their localization in the allergen does not necessarily coincide. In the case of the T-cell receptor, only the linear amino acid sequence is important for recognition [77]. In contrast, B-cell epitopes recognized by IgE antibodies are either linear or conformational and are located on the surface of the molecule accessible to antibodies. Thus, conformational B-cell epitopes require a proper folded allergen for efficient binding of inhaled allergens [78]. The extension of the epitope may range from 5 to 8 or longer amino acids for IgE to be able of binding to the epitope [79], [80], [99].

Molecular modeling and sequence polymorphisms characterization help identifying specific regions, which could be candidates for the development of peptide-based immunotherapeutic reagents for pollen allergy as has already been described for other allergens [8], [69], [74], [81], while conserved regions could be responsible of the cross-reaction between pollen and plant derived food allergen [82]. Epitopes prediction based on knowledge derived from structural surface features such as increased solvent accessibility [83], backbone flexibility [84], and hydrophilicity [85] were found to correlate well with antigenicity in the present study. We have identified surface patterns (conformational epitopes), as well as multiple regions (B- and T-cell epitopes) in the olive profilins, exhibiting differences in length and variability (Table 2, Table 3, Table 4), depicted in the surface of the allergens Ara t 8, Bet v 2 or Hev b 8 to show their distribution (Figure 5, Figure 6). In addition, we have found shared common B- and T-cell epitopes among cultivars and between species, in addition to epitopes differentially distributed in specific cultivars and species (Figure 6, Table 2, Table 3).

Furthermore, we found an extensive correlation between conformational and B- and T-cell epitopes in olive profilins, in addition to high variability in their sequences (Table 5, Table S5). Furthermore, we have identified conformational epitopes in *Olea europaea* L. that specifically overlap only with T-cells, i.e. C8(P79), C7(Q79), or with B-cells, i.e. E1(T5), thus likely playing a fundamental role in pollen allergen cross-reactivity.

Linear B- and/or T- cell epitopes may play most important roles in cross-reactivity between food allergens, and between pollen and food allergens [86], since food processing or digestion may increase the number or the accessibility of IgE binding epitopes. Thus, Bet v 1-related food allergens have been described to led to a loss of some or all the B-cell epitopes (but not the T-cell epitopes) by denaturalization/digestion [87].

Our study has identified commonly shared conformational B-cell epitopes in olive (i.e. epitope 1, epitope 2 from Table 5), which may play an important role in broad cross-reactivity between pollen allergens of different non-related species. In addition, olive conformational-T-cell epitopes such as 5, 7, and 8 (Table 5) may be involved in pollen and/or pollen-food allergens wide cross-reactivity. The variability in their surface residues might contribute to generate areas of the protein enable of being differentially recognized as Th_2_- inducing antigens. Depending on the location of these polymorphic residues, recognition by IgE/IgG may be also affected (i.e. nine Bet v 1 isoforms, sharing an average identity of 84–99%, displayed different allergenic properties both *in vivo* and *in vitro*) [88].

Furthermore, it is commonly found at structural level the presence of antigenic determinants integrated in 2-D structure elements, which protrude from the surface of the protein, such as coils and loops [69]. Our results have shown that among the three characteristic loops that distinguish plant profilins from other species [10], the first loop situated between N-terminal α-helix 1 and β-strand 1 of several olive profilin sequences contains an insertion of three to six residues, in addition to multiple micro-heterogeneities [8, current study]. These features have been also found in profilins of *Betula pendula*, *Corylus avellana*, *Phleum pratense*, *Zea mays* [8], current study and other plant species [65], [66]. The length of these structural elements together with the different degree of variability might be responsible of increasing even more the variability of molecular epitopes among olive cultivars, and between species. These differences may additionally increase the differences and the extension of the allergenic reactions [8], [68], [69], [89]. Differences in antigenic determinants localized in structural loops have been shown previously in the Amb t 5 allergen, which exhibited an immune-dominant B-cell epitope located in the loop 3, responsible for large number of allergy responses [90]. Other similar examples are the major IgE-binding regions inter-helix loop of the allergen Pru p 3 [91], or the area integrated by a P-loop in Bet v 1 [92], which was also found in the Bet v 1 homologous food allergens [93].

Olive profilins also exhibited two additional loops (between β-strands 4 and 5 and between β-strands 5 and 6), both of them taking part of the actin binding surface, situated in the plant profilin specific solvent-filled pocket. These two loops and their variability might be also responsible for immune cross-reactivity between human and plant profilins in atopic patients [64], since timothy-grass and human profilin exhibit a high similarity in these two structural loops [9].

Multimeric forms of allergens have been shown as an additional structural factor responsible for the presence of cross-reactions among profilins from several species [94]. Dimeric or oligomeric forms of allergens may favor cross-linking compared to monomers due to an effective increase of the number of epitopes. Birch profilin has been described to induce an IgG-subclass2 (IgG2) in mouse and primates, which is considered a typical response to polymeric antigens [95]. Recombinant maize pollen profilin isoform 1 (ZmPRO1) forms multimeric structures [94], which are resistant to denaturation and to the action of reducing agents, similarly to human profilin purified from platelets [48]. Oligomerization of profilin has been described also in naturally isolated and recombinant mugwort pollen profilin, indicating that the solutions of this protein contains dimeric and tetrameric forms stabilized by disulphide bridges and/or ionic interactions [96]. Pollen profilin released into the extracellular space, normally finds favorable physico-chemical conditions (such as red-ox state of the respiratory tract mucosa) that promote the presence of polymeric forms [94], [97].

In our study, we have pointed out the possible existence of dimeric forms of profilin, where cysteine bridges may be involved in the structural stabilization. Limited data is available about biochemical and immunologic significance of the formation of profilin multimers. Dimeric and tetrameric forms do not significantly differ in their ability to bind serum IgE from pollen allergic patients [96]. However, multimeric forms may be able to bind to a high number of IgE antibodies than monomers, making oligomeric forms of profilin more allergenic, exhibiting strong reactions compared to monomers as a result of a larger molecules surface containing additional epitopes for IgE-mediated histamine release. Thus, multimeric forms of profilins might constitute an additional mechanism of increasing the number of epitopes and variability. Furthermore, the differential recognition of plant profilin multimeric forms by immune system would not be a consequence of a simple additive effect. Otherwise multimeric forms of profilin may operate synergically to facilitate the access of IgEs to defined epitopes in this big macromolecular complex.

## Methods

### Profilin sequences

GenBank/EMBL Database entries of previously cloned pollen profilins and sequences from *Olea europaea* L. (24 cultivars), *Betula pendula*, *Corylus avellana*, *Phleum pratense*, and *Zea mays* [8] were retrieved from Uniprot database (www.uniprot.org), and used for the present study.

### Phylogenetic analysis of profilin sequences

Protein sequences from the five plant species were used to perform three different phylogenetic analyses. These groups of profilin sequences were made according to the structural template (PDB numbers 1cqa, 1g5uA or 3nul) more suitable for each sequence.

Sequences alignments were performed by using ClustalW multiple sequence alignment tool (www.ebi.ac.uk/Tools/clustalw). These alignments were created using the Blosum62 matrix, multiple alignment gap opening/extension penalties of 10/0.5 and pairwise gap opening/extension penalties of 10/0.1. The outputs were manually checked to optimize the alignment by using Bioedit (www.mbio.ncsu.edu/bioedit/bioedit.html). Phylogenetic trees were generated by the neighbor-joining method (NJ), and the branches were tested with 1000 bootstrap replicates. Trees were visualized using Treedyn (www.treedyn.org).

### Template assessment

All profilin sequences were searched for homology in the Protein Data Bank (PDB). Homologous templates suitable for profilins were selected by using Swiss-Prot database and template assessment (swissmodel.expasy.org) and BLAST server (ncbi.nlm.nih.gov/). The BioInfoBank Metaserver (meta.bioinfo.pl) which employs fold recognition for homology search was also used for template selection. The crystal structure of template was retrieved from PDB (1cqa, 1g5uA and 3nul) and used for homology modeling.

### Homology modeling

Sequences were modeled through SWISS-MODEL via the ExPASy web server (swissmodel.expasy.org), by using the top PDB closest template structures previously assessed. An initial structural model was generated for the different profilin sequences and checked for recognition of errors in 3D structures using ProSA (prosa.services.came.sbg.ac.at/prosa.php), and for a first overall quality estimation of the model with QMEAN (swissmodel.expasy.org/qmean/cgi/index.cgi).

Final structures were subjected to energy minimization with GROMOS96 force field energy implemented in Deep-View/Swiss-PDBViewer v3.7 (spdbv.vital-it.ch) to improve the van der Waals contacts and correct the stereochemistry of the model. For each sequence analyzed, the quality of the model was assessed by QMEAN, checking proteins stereology with PROCHECK (www.ebi.ac.uk/thornton-srv/software/PROCHECK), ProSA programs, as well as the protein energy with ANOLEA (protein.bio.puc.cl/cardex/servers/anolea). The Ramachandran plot for the models was generated, showing the majority of the protein residues in the favored regions.

### Structural comparison and evolutionary conservational analysis

Protein models were superimposed on the template crystal structures to calculate average distance between their Cα backbones. The 2-D protein structural analysis, protein superimpositions and surface protein contours analysis were performed and visualized in PyMol software (www.pymol.org).

Recognition of profilin secondary structural elements was assessed by Segmer algorithm [98], which threads sequence segments through the Protein Data Bank (PDB) library (www.pdb.org) to identify conserved substructures. Furthermore, elements of the secondary structure were also identified, and compared with the results obtained with other different approaches: SSpro8 (Scratch Protein Predictor), which adopts the full DSSP 8-class output classification (scratch.proteomics.ics.uci.edu), NetSurfP ver. 1.1 (www.cbs.dtu.dk), and PSIPRED (http://bioinf.cs.ucl.ac.uk/psipred) fold servers.

Prediction and confirmation of plant specific binding pocket localization in the 3D models was performed by using PocketFinder (www.modelling.leeds.ac.uk/pocketfinder/help.html) and LIGSiteCSC (scoppi.biotech.tu-dresden.de/pocket) software.

Disulphide bridges formation, number and red-ox estate were analyzed with the DIpro software (scratch.proteomics.ics.uci.edu). The distances between every two α carbons (Cα) from all cysteines were measured by using the DeepView/Swiss-PDBViewer v3.7 software (spdbv.vital-it.ch).

Protein models for profilin proteins were submitted to ConSurf server (consurf.tau.ac.il) in order to generate evolutionary related conservation scores, helping us to identify functional region in the proteins. Functional and structural key residues in the profilin sequences were confirmed by ConSeq server (conseq.tau.ac.il).

### Solvent accessible surface area and Poisson–Boltzmann electrostatic potential

Solvent accessible surface area (SASA), defined as the percentage of surface area of a biomolecule that is accessible to a solvent for each residue was calculated by using the GETAREA v1.1 program (curie.utmb.edu/getarea.html). Relative values were calculated in relation to the average SASA of the respective residue in the peptide GXG, being X each amino acid of the profilin sequence.

The electrostatic Poisson-Boltzmann (PB) potentials for the structures were obtained using APBS molecular modeling software implemented in PyMol 0.99 (www.pymol.org) with AMBER99 to assign the charges and radii to all the atoms (including hydrogens), and optimized with the Python software package PDB2PQR. Fine grid spaces of 0.35 Å were used to solve the linearized PB equation in sequential focusing multigrid calculations in a mesh of 130 points per dimension at 310.00 K. The dielectric constants were 2.00 for the proteins and 80.00 for water. The output mesh was processed in the scalar OpenDX format to render the isocontours and maps on the surfaces with PyMOL 0.99. Potential values are given in units of kT per unit charge (k Boltzmann's constant; T temperature).

### Allergenicity profile assessment

Allergenicity of profilin sequences was checked by a full FASTA alignment in the Structural Database of Allergenic Proteins (SDAP) (Fermi.utmb.edu/SDAP). Allergenicity profile was assessed by combination of different parameters: hydrophobicity, antigenicity and SASA. This last was compared to absolute surface area (ASA) of each residue calculated by DSSP program (swift.cmbi.ru.nl/gv/dssp). These values were transformed to relative values of ASA and visualized by ASAView (www.netasa.org/asaview).

### Ole e 2 B-cell epitopes analysis

Changes in the amino acid sequences of the epitopic immune-dominant regions to be recognized by IgG and IgE immunoglobulins (B-cell epitopes) were meticulously analyzed for all the profilin sequences of the five plant species through comparison with 5 epitopes called 10A4, 5F2, 9A7, 9G4 and 3H8, previously characterized in recombinant profilin Hel a 2 from sunflower by using overlapping synthetic peptides and monoclonal antibodies [99].

### Ole e 2 T-cell epitopes identification and analysis

The identification of MHC Class-II binding regions in the antigen sequences for all the profilin sequences of the five plant species was performed by using neuronal networks and quantitative matrices derived from published literature. Promiscuous peptides binding to multiple HLA class II molecules were selected. The analysis was made by using the TEPITOPE software (www.bioinformation.net/ted), with a threshold of 5% for the most common human HLA-DR alleles [DRB1*0101 (DR1), DRB1*0301(DR3), DRB1*0401 (DR4), DRB1*0701 (DR7), DRB1*0801(DR8), DRB1*1101 (DR5) and DRB1*1501 (DR2)] among Caucasian population, and covering a large proportion of the peptides that bind with human HLA.

### Ole e 2 conformational epitopes identification and analysis

The structure of the allergen Hev b 8 from *Hevea brasiliensis* latex profilin (Q9LEI8, model 1g5uA), Bet v 2 from *Betula pendula* (Uniprot accession number P25816, model 1cqa), and Ara t 8 from *Arabidopsis thaliana* (Q42449, model 3nul) (www.pdb.org), were used to predict amino acid residues forming potential conformational epitopes on the surface of the protein. Relative values were calculated in relation to the average SASA of the respective residue in the peptide GXG. The distances between residues were calculated using the Swiss-PDB Viewer (spdbv.vital-it.ch). Residues contributing to conformational epitopes were predicted by a structure-based algorithm, which is a modification of a previously published method [100]. Briefly: i) Protruding residues with relative SASA>75% were chosen as center of the epitopes. If two or more of these residues were closer to each other than 0.5 nm, they were combined to form a single epitope. ii) For each one of these residues, all partially accessible residues (SASA>20%) within 1 nm distance from the central residue(s) were included in the epitope. The chosen epitope radius reflects the typical size of an antigen - antibody contact surface [101]. iii) Finally, single residues located in the linear sequence between two residues or stretches that were already part of the epitope were also included. The reason behind this step to be included was the assumption that an amino acid will significantly affect the conformation of its neighbors.

## Supporting Information

Figure S1 **Diagram representation of highly antigenic regions within profilin protein sequences of the five species studied.** Eight areas of high antigenicity are highlighted with red (shared) and blue (no shared) colored boxes for olive and birch profilins, and seven areas for the profilins of the rest of the species, as a result of the combination of parameters such as A) hydrophobicity (or hydrophilicity), Kyte-Doolitte scale, B) antigenicity, Welling method, C) antigenicity, Parker method, D) 2-D structural elements. Surface accessibility of amino acids (SASA>25%) (discontinue red line in the left, central and right panels, respectively, of the Figure 2a) were used as another parameter to delimit areas of high antigenicity. Reference sequences are these chosen as reference in the alignments of profilin proteins for each specie: DQ138336 for *Olea europaea* L., M65179 for *Betula pendula*, DQ663544 for *Corylus avellana*, DQ663535 for *Phleum pratense*, and DQ663560 for *Zea mays*.
(TIF)

Figure S2 **Conformational epitope 1.** Amino acids integrating the conformational epitope 1 were depicted in red color over the surface of the 3nul structural model. Green color represents additional amino acids that belong to the plant characteristic loop.
(TIF)

Table S1 **Template assessment for profilin protein sequences.** Profilin sequences corresponding to 24 cultivars of A) *Olea europaea* L., and the species B) *Betula pendula*, *Corylus avellana*, *Phleum pratense* and *Zea mays*, were assessed in order to determine the best crystallographic model that fit with every single sequence of profilin. Table includes parameters such as highest % of identity (73–93%), and best E-value.
(DOCX)

Table S2 **Conservational analysis of key amino acids implicated in profilin folding and 3D structure maintenance.** Residues which percentage of conservation was different that 100% were highlighted in bold and grey shadowed.
(DOCX)

Table S3 **Disulfide bridges analysis.** A) Distance (Å) between every pair of cysteines (C α ) in the sequences of profilins, calculated using the program DeepView/Swiss PDB Viewer v3.7. Identifies pair of cysteines most likely to form disulfide bridges were highlighted in bold. B) Distances (Å) between Cα of possible inter-catenaries cysteine bridges of profilin which could form dimers. Those cysteines most likely to form disulfide bridges are in bold and larger. Identifies pair of cysteines most likely helping to form profilin dimmers were highlighted in bold.
(DOCX)

Table S4 **Conservational analysis of residues implicate in PIP-binding domains.** A) Residues which percentage of conservation is lower that 100% were highlighted in bold and grey shadowed. Species of *Betulaceae* and *Poaceae* genus seem to be the most variable species. B) Examples of punctual changes in the sequence of different profilins from the five species analyzed. *Olea europaea* L. is the specie with more number of sequences changed.
(DOCX)

Table S5 **Conformational epitopes analysis.** The analysis was performed for profilin sequences corresponding to A) *Olea europaea* L., B) *Betula pendula*, C) *Corylus avellana*, D) *Phleum pratense*, and E) *Zea mays*. Central residue of conformational epitopes was pointed out with a box. B-cell epitopes partially or totally overlapping with conformational epitopes were highlighted with a color code: red for 10A4, green for 5F2, blue for 9A7, yellow for 9G4, and pink for 3H8. T-cell epitopes partially or totally overlapping with conformational epitopes were highlighted with grey shadows.
(DOCX)
