# Correction: Analysis of the Effects of Polymorphism on Pollen Profilin Structural Functionality and the Generation of Conformational, T- and B-Cell Epitopes

**DOI:** 10.1371/annotation/3008c3af-753f-4085-b85e-5ae0c34a7889

**Published:** 2014-01-02

**Authors:** Jose C. Jimenez-Lopez, María I. Rodríguez-García, Juan D. Alché

The legends for Figures 1 and 2 were incorrectly switched. The legend that appears for Figure 1 should be for Figure 2, and the legend that appears for Figure 2 should be for Figure 1. Additionally, the legends for Figures 3 and 4 were incorrectly switched. The legend that appears for Figure 3 should be for Figure 4, and the legend that appears for Figure 4 should be for Figure 3. 

