# Supplementary figures and images for: Analysis of the Effects of Polymorphism on Pollen Profilin Structural Functionality and the Generation of Conformational, T- and B-Cell Epitopes
Source: PLoS One. 2013 Oct 17;8(10):e76066. doi: 10.1371/journal.pone.0076066 (PMC3798325; doi:10.1371/journal.pone.0076066)

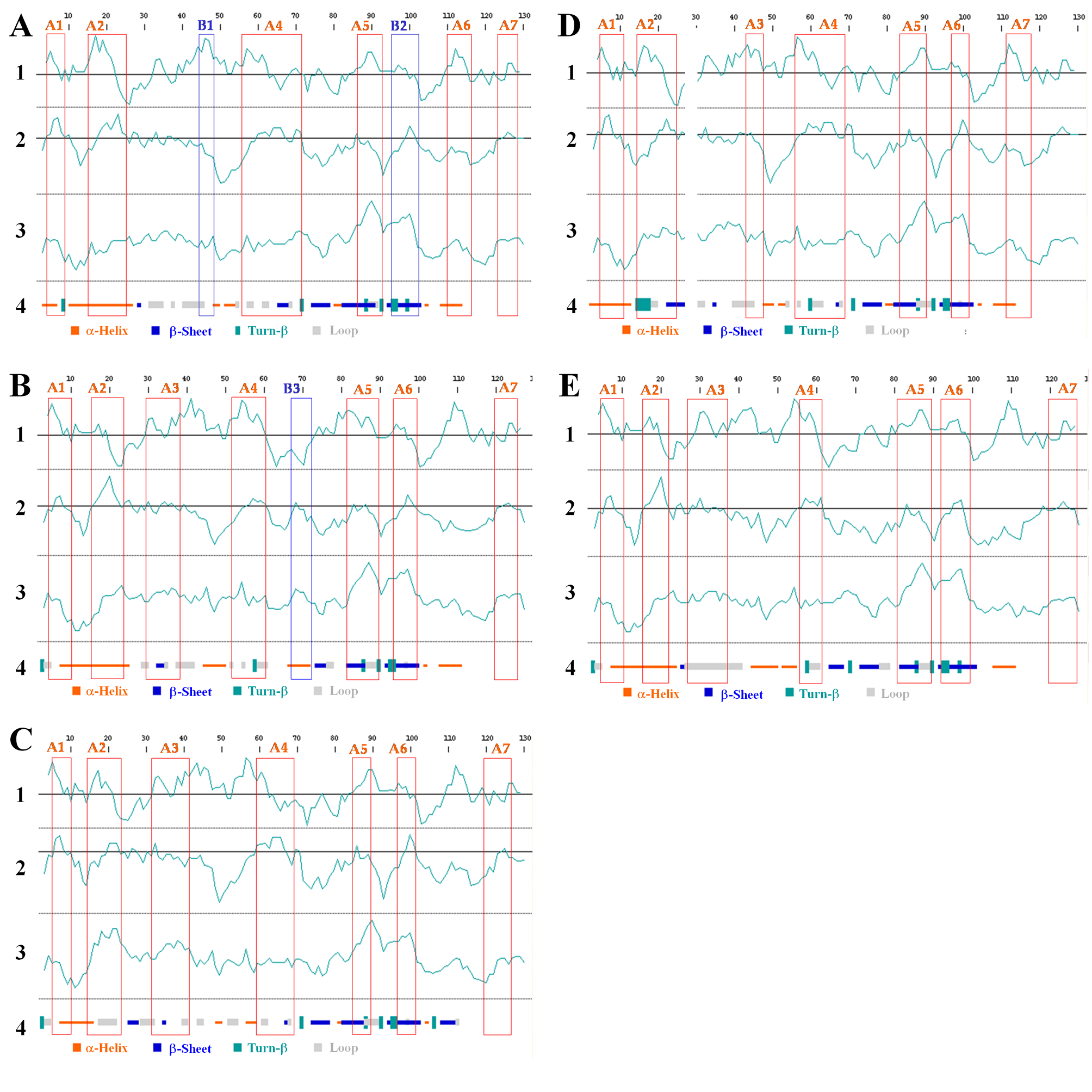

Supplement: Figure S1 — Diagram representation of highly antigenic regions within profilin protein sequences of the five species studied. Eight areas of high antigenicity are highlighted with red (shared) and blue (no shared) colored boxes for olive and birch profilins, and seven areas for the profilins of the rest of the species, as a result of the combination of parameters such as A) hydrophobicity (or hydrophilicity), Kyte-Doolitte scale, B) antigenicity, Welling method, C) antigenicity, Parker method, D) 2-D structural elements. Surface accessibility of amino acids (SASA>25%) (discontinue red line in the left, central and right panels, respectively, of the Figure 2a) were used as another parameter to delimit areas of high antigenicity. Reference sequences are these chosen as reference in the alignments of profilin proteins for each specie: DQ138336 for Olea europaea L., M65179 for Betula pendula, DQ663544 for Corylus avellana, DQ663535 for Phleum pratense, and DQ663560 for Zea mays. (TIF) [file pone.0076066.s001.tif]

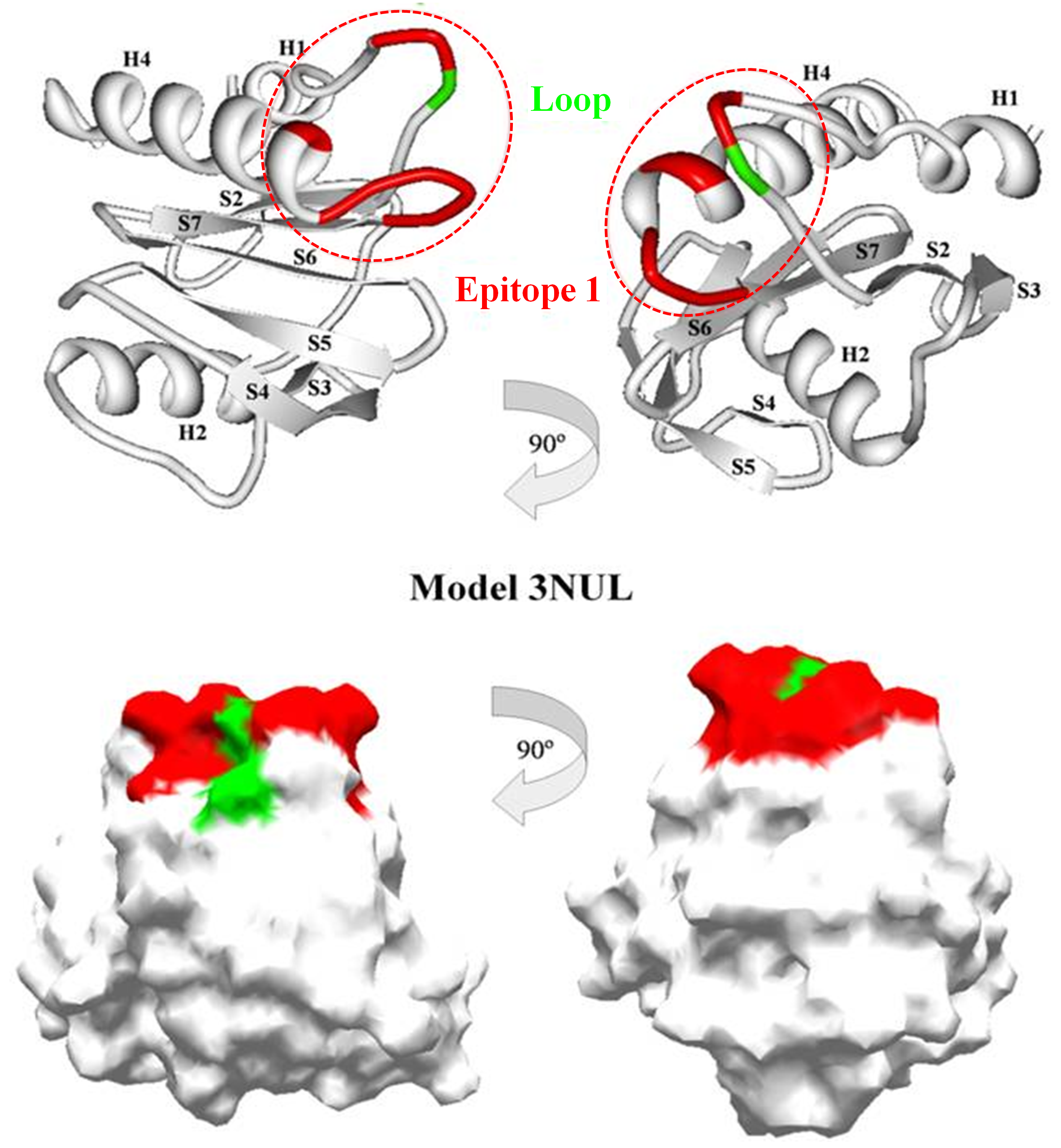

Supplement: Figure S2 — Conformational epitope 1. Amino acids integrating the conformational epitope 1 were depicted in red color over the surface of the 3nul structural model. Green color represents additional amino acids that belong to the plant characteristic loop. (TIF) [file pone.0076066.s002.tif]
