# Supplementary material for: Analysis of the Effects of Polymorphism on Pollen Profilin Structural Functionality and the Generation of Conformational, T- and B-Cell Epitopes
Source: PLoS One. 2013 Oct 17;8(10):e76066. doi: 10.1371/journal.pone.0076066 (PMC3798325; doi:10.1371/journal.pone.0076066)
Supplement: Table S1 — Template assessment for profilin protein sequences. Profilin sequences corresponding to 24 cultivars of A) Olea europaea L., and the species B) Betula pendula, Corylus avellana, Phleum pratense and Zea mays, were assessed in order to determine the best crystallographic model that fit with every single sequence of profilin. Table includes parameters such as highest % of identity (73–93%), and best E-value. (DOCX) [file pone.0076066.s003.docx]

**Table S1**

**A)**

| **GeneBank**  **accession N°** | ***Olea europaea* L.**  **Cultivar** | **PDB**  **model** | **Profilin**  **(SDAP)** | **Identity (%)** | **E-value** |
| --- | --- | --- | --- | --- | --- |
| **Y12425** | **-**  **-**  **-** | **1CQA** | **Bet v 2** | **81** | **2.76 e-56** |
| **Y12429** |  |  |  | **82** | **3.86 e-56** |
| **Y12430** |  |  |  | **81** | **1.09 e-55** |
| **DQ138355** | **Acebuche** | **1G5Ua** | **Hev b 8** | **80** | **2.36 e-59** |
| **DQ138356** |  |  |  |  | **2.36 e-59** |
| **DQ138357** |  |  |  |  | **1.24 e-58** |
| **DQ138327** | **Arbequina** | **1G5Ua** | **Hev b 8** | **81** | **6.97 e-60** |
| **DQ138328** |  |  |  | **80** | **2.3 6e-59** |
| **DQ138329** |  | **1CQA** | **Bet v 2** | **81** | **1.19 e-55** |
| **DQ138330** |  |  |  | **82** | **3.86 e-56** |
| **DQ317563** | **Bella de España** | **1CQA** | **Bet v 2** | **81** | **2.44 e-56** |
| **DQ317564** |  |  |  | **81** | **3.46 e-56** |
| **DQ640909** |  | **1G5Ua** | **Hev b 8** | **74** | **4.93 e-57** |
| **DQ640910** |  |  |  | **88** | **5.31e-63** |
| **DQ138335** | **Blanqueta** | **1CQA** | **Bet v 2** | **81** | **2.86 e-56** |
| **DQ138338** |  |  |  | **83** | **1.24 e-56** |
| **DQ138336** |  | **3NUL** | **Ara t 8** | **77** | **1.92e-53** |
| **DQ138337** |  | **1G5Ua** | **Hev b 8** | **80** | **4.02 e-59** |
| **DQ138331** | **Cornicabra** | **1G5Ua** | **Hev b 8** | **79** | **2.04 e-58** |
| **DQ138332** |  |  |  | **80** | **3.49e-59** |
| **DQ138333** |  |  |  |  | **2.36 e-59** |
| **DQ138334** |  | **1CQA** | **Bet v 2** | **81** | **4.30 e-55** |
| **DQ138342** | **Empeltre** | **1CQA** | **Bet v 2** | **82** | **3.86 e-56** |
| **DQ138343** |  |  |  |  | **3.86ce-56** |
| **DQ138344** |  | **1G5Ua** | **Hev b 8** | **80** | **6.05 e-59** |
| **DQ317565** | **Farga** | **1CQA** | **Bet v 2** | **82** | **3.86 e-56** |
| **DQ317566** |  | **1G5Ua** | **Hev b 8** | **80** | **2.36 e-59** |
| **DQ317567** |  | **1CQA** | **Bet v 2** | **81** | **3.79 e-55** |
| **DQ317568** | **Frantoio** | **1G5Ua** | **Hev b 8** | **81** | **6.97 e-60** |
| **DQ317569** |  | **1CQA** | **Bet v 2** | **83** | **2.50 e-56** |
| **DQ317570** | **Galega** | **1G5Ua** | **Hev b 8** | **80** | **4.02 e-59** |
| **DQ061979** | **Hojiblanca** | **1CQA** | **Bet v 2** | **81** | **3.58 e-56** |
| **DQ061980** |  |  |  | **82** | **3.86 e-56** |
| **DQ061981** |  |  |  |  |  |
| **DQ061982** |  | **1G5Ua** | **Hev b 8** | **80** | **1.44 e-58** |
| **DQ138345** | **Leccino** | **1G5Ua** | **Hev b 8** | **81** | **1.67 e-59** |
| **DQ138346** |  |  |  | **81** | **6.97 e-60** |
| **DQ138347** |  |  |  | **80** | **3.95 e-58** |
| **DQ317571** | **Lechín de Granada** | **1CQA** | **Bet v 2** | **82** | **3.86 e-56** |
| **DQ317572** |  | **1G5Ua** | **Hev b 8** | **80** | **1.37 e-58** |
| **DQ640906** |  |  |  | **75** | **4.39 e-57** |
| **DQ028766** | **Lechín de Sevilla** | **1CQA** | **Bet v 2** | **82** | **3.86 e-56** |
| **DQ061978** |  |  |  | **83** | **2.10e-56** |
| **DQ061976** |  | **1G5Ua** | **Hev b 8** | **80** | **4.37 e-59** |
| **DQ061977** |  |  |  |  | **5.95 e-59** |
| **DQ138339** | **Loaime** | **1CQA** | **Bet v 2** | **82** | **1.27 e-56** |
| **DQ138340** |  |  |  |  |  |
| **DQ138341** |  |  |  |  |  |
| **DQ640903** |  | **3NUL** | **Ara t 8** | **78** | **9.42 e-54** |
| **DQ138362** | **Lucio** | **1CQA** | **Bet v 2** | **82** | **3.86 e-56** |
| **DQ138365** |  |  |  |  |  |
| **DQ138363** |  | **1G5Ua** | **Hev b 8** | **80** | **3.95 e-59** |
| **DQ138364** |  |  |  |  | **5.20 e-59** |
| **DQ640908** |  |  |  | **88** | **6.12 e-64** |
| **DQ117911** | **Manzanilla Sevilla** | **1CQA** | **Bet v 2** | **83** | **3.70 e-57** |
| **DQ138325** |  |  |  | **82** | **3.86e-56** |
| **DQ138326** |  |  |  | **81** | **3.58 e-56** |
| **DQ138324** |  | **1G5Ua** | **Hev b 8** | **80** | **9.81 e-60** |
| **DQ317573** | **Morrut** | **1G5Ua** | **Hev b 8** | **80** | **1.34 e-58** |
| **DQ317574** |  |  |  |  | **6.80 e-58** |
| **DQ317575** |  |  |  |  | **1.29 e-59** |
| **DQ317576** |  |  |  |  | **1.44 e-58** |
| **DQ317580** | **Picual** | **1G5Ua** | **Hev b 8** | **80** | **2.60 e-59** |
| **DQ317581** |  |  |  | **81** | **6.97 e-60** |
| **DQ317582** |  |  |  |  | **6.97 e-60** |
| **DQ640904** |  |  |  | **78** | **1.06e-58** |
| **DQ663558** |  |  |  |  | **8.99 e-60** |
| **DQ663553** |  | **3NUL** | **Ara t 8** | **75** | **1.76 e-53** |
| **DQ663554** |  |  |  | **76** | **3.66 e-53** |
| **DQ663555** |  |  |  | **73** | **7.01 e-52** |
| **DQ663556** |  |  |  | **75** | **2.18 e-53** |
| **DQ663557** |  |  |  | **75** | **1.33 e-53** |
| **DQ117907** | **Picudo** | **1G5Ua** | **Hev b 8** | **80** | **6.97 e-59** |
| **DQ117909** |  |  |  | **80** | **2.36 e-59** |
| **DQ117910** |  |  |  | **79** | **3.13 e-58** |
| **DQ117908** |  | **1CQA** | **Bet v 2** | **82** | **2.42 e-55** |
| **DQ138348** | **Sevillenca** | **1G5Ua** | **Hev b 8** | **79** | **1.13 e-58** |
| **DQ138349** |  |  |  | **81** | **4.91 e-60** |
| **DQ138350** |  |  |  | **80** | **2.36 e-59** |
| **DQ317577** | **Sourani** | **1G5Ua** | **Hev b 8** | **80** | **2.36 e-59** |
| **DQ317578** |  |  |  |  | **4.40 e-59** |
| **DQ317579** |  |  |  |  | **1.17 e-58** |
| **DQ640905** |  |  |  | **88** | **3.93 e-63** |
| **DQ117902** | **Verdial Huevar** | **1G5Ua** | **Hev b 8** | **81** | **2.93 e-59** |
| **DQ117903** |  | **1CQA** | **Bet v 2** | **82** | **3.86 e-56** |
| **DQ117904** |  |  |  | **83** | **3.70 e-57** |
| **DQ117905** |  |  |  | **82** | **1.27 e-56** |
| **DQ117906** |  |  |  | **82** | **5.25 e-57** |
| **DQ138358** | **Verdial Málaga** | **1CQA** | **Bet v 2** | **80** | **9.42 e-55** |
| **DQ138359** |  |  |  | **82** | **3.86 e-56** |
| **DQ138360** |  |  |  | **83** | **1.11 e-56** |
| **DQ138361** |  |  |  | **80** | **3.35 e-55** |
| **DQ138351** | **Villalonga** | **1G5Ua** | **Hev b 8** | **80** | **1.57 e-58** |
| **DQ138352** |  |  |  |  | **1.88 e-58** |
| **DQ640907** |  |  |  | **78** | **1.06 e-58** |
| **DQ138353** |  | **1CQA** | **Bet v 2** | **81** | **1.27 e-55** |
| **DQ138354** |  |  |  | **80** | **1.88 e-58** |

**B**)

| **GeneBank**  **accession N°** | ***Olea europaea* L.**  **Cultivar** | **PDB**  **model** | **Profilin**  **(SDAP)** | **Identity (%)** | **E-value** |
| --- | --- | --- | --- | --- | --- |
| **M65179** | ***Betula pendula*** | **1CQA** | **Bet v 2** | **92** | **9.35 e-61** |
| **DQ650633** |  |  |  | **93** | **4.52 e-61** |
| **DQ663543** | ***Corylus avellana*** | **1CQA** | **Bet v 2** | **88** | **5.53 e-58** |
| **DQ663544** |  |  |  |  | **1.41 e-57** |
| **DQ663546** |  |  |  |  | **5.53 e-58** |
| **DQ663548** |  |  |  |  | **5.22 e-58** |
| **DQ663549** |  |  |  | **90** | **2.52 e-59** |
| **DQ663550** |  |  |  |  |  |
| **DQ663551** |  |  |  |  |  |
| **DQ663552** |  |  |  |  |  |
| **DQ663545** |  | **1G5Ua** | **Hev b 8** | **86** | **2.34 e-62** |
| **DQ663547** |  | **3NUL** | **Ara t 8** | **73** | **6.84 e-52** |
| **Y09457** | ***Phleum pratense*** | **3NUL** | **Ara t 8** | **75** | **7.69 e-53** |
| **Y09456** |  |  |  | **76** | **8.78 e-54** |
| **X77583** |  |  |  | **77** | **2.97 e-54** |
| **Y09458** |  |  |  |  |  |
| **DQ663535** |  |  |  | **75** | **1.76 e-53** |
| **DQ663536** |  |  |  |  | **1.00 e-53** |
| **DQ663539** |  |  |  |  | **1.76 e-53** |
| **DQ663540** |  |  |  |  | **1.32 e-53** |
| **DQ663541** |  |  |  | **74** | **3.60 e-53** |
| **DQ663542** |  |  |  |  | **6.29 e-53** |
| **DQ663537** |  | **1G5Ua** | **Hev b 8** | **76** | **2.45 e-58** |
| **DQ663538** |  |  |  |  | **3.71 e-58** |
| **X73279** | ***Zea mays*** | **1G5Ua** | **Hev b 8** | **82** | **6.82 e-61** |
| **X73280** |  |  |  | **78** | **1.16 e-59** |
| **X73281** |  |  |  | **79** | **1.35 e-59** |
| **DQ663565** |  |  |  | **78** | **8.99 e-60** |
| **DQ663559** |  |  |  | **79** | **2.20 e-59** |
| **DQ663561** |  |  |  |  | **8.84 e-60** |
| **DQ663564** |  |  |  |  | **1.65 e-59** |
| **DQ663560** |  |  |  | **80** | **9.45 e-61** |
| **DQ663562** |  |  |  | **81** | **6.38 e-61** |
| **DQ663563** |  |  |  | **82** | **3.30 e-61** |
