# Supplementary material for: Analysis of the Effects of Polymorphism on Pollen Profilin Structural Functionality and the Generation of Conformational, T- and B-Cell Epitopes
Source: PLoS One. 2013 Oct 17;8(10):e76066. doi: 10.1371/journal.pone.0076066 (PMC3798325; doi:10.1371/journal.pone.0076066)
Supplement: Table S2 — Conservational analysis of key amino acids implicated in profilin folding and 3D structure maintenance. Residues which percentage of conservation was different that 100% were highlighted in bold and grey shadowed. (DOCX) [file pone.0076066.s004.docx]

**Table S2**

| **Amino acid**  (sequence position) | ***Olea europaea*** | ***Betula pendula*** | ***Corylus avellana*** | ***Phleum pratense*** | ***Zea mays*** |
| --- | --- | --- | --- | --- | --- |
| W(3) | 100 | 100 | 100 | 100 | 100 |
| Y(6) | 100 | 100 | 100 | 100 | 100 |
| D(8) | 100 | 100 | 100 | 100 | 100 |
| A (23, 25, 26) | **99** | 100 | **20** | 100 | 100 |
| A (24, 26, 27) | 100 | - | 100 | 100 | 100 |
| I (25, 27, 28) | **99** | 100 | 100 | 100 | 100 |
| G (27, 29, 30) | 100 | 100 | 100 | 100 | 100 |
| W (33, 35, 36) | 100 | 100 | 100 | 100 | 100 |
| A (34, 36, 37) | 100 | 100 | 100 | 100 | 100 |
| E (46, 48, 49) | 100 | 100 | 100 | 100 | 100 |
| G (64, 66, 67) | 100 | 100 | **90** | 100 | 100 |
| G (69, 71, 72) | **90** | 100 | 100 | **30** | **10** |
| K (71, 73, 74) | 100 | 100 | 100 | 100 | 100 |
| K (86, 88, 89) | 100 | 100 | 100 | 100 | 100 |
| T (97, 99, 100) | 100 | 100 | 100 | 100 | 100 |
| G (113, 115, 116) | 100 | 100 | 100 | 100 | 100 |
| Y (125, 127, 128) | 100 | 100 | 100 | 100 | 100 |
| L (126, 128, 129) | 100 | 100 | 100 | 100 | 100 |
