# Supplementary material for: Analysis of the Effects of Polymorphism on Pollen Profilin Structural Functionality and the Generation of Conformational, T- and B-Cell Epitopes
Source: PLoS One. 2013 Oct 17;8(10):e76066. doi: 10.1371/journal.pone.0076066 (PMC3798325; doi:10.1371/journal.pone.0076066)
Supplement: Table S3 — Disulfide bridges analysis. A) Distance (Å) between every pair of cysteines (C α ) in the sequences of profilins, calculated using the program DeepView/Swiss PDB Viewer v3.7. Identifies pair of cysteines most likely to form disulfide bridges were highlighted in bold. B) Distances (Å) between Cα of possible inter-catenaries cysteine bridges of profilin which could form dimers. Those cysteines most likely to form disulfide bridges are in bold and larger. Identifies pair of cysteines most likely helping to form profilin dimmers were highlighted in bold. (DOCX) [file pone.0076066.s005.docx]

**Table S3**

**A)**

| **Model** | ***Olea europaea* L.** | | | | | ***Betula pendula*** | ***Corylus avellana*** | | ***Phleum pratense*** | ***Zea mays*** |
| --- | --- | --- | --- | --- | --- | --- | --- | --- | --- | --- |
|  | Two cysteins  (Å) | | Three cysteins  (Å) | | | Two cysteins  (Å) | | | | |
| Cystin position | **C^13^-C^115^** | **C^13^-C^118^** | **C^13^-C^106^** | **C^13^-C^118^** | **C^106^-C^118^** | **C^13^-C^117^** | **C^13^-C^115^** | **C^13^-C^117^** | **C^13^-C^115^** | **C^13^-C^115^** |
| 1g5u | 10.41 | **8.64** | 12.45 | **8.64** | 11.19 | 11.28 | 10.41 | 11.28 | 10.41 | 10.41 |
| 3nul | 10.46 | **8.09** | 12.23 | **8.09** | 10.89 | 11.19 | 10.46 | 11.19 | 10.46 | 10.46 |
| 1a0k | 10.62 | **8.10** | 12.46 | **8.10** | 10.89 | 11.20 | 10.62 | 11.20 | 10.62 | 10.62 |

**B)**

| Model 1g5u | **Inter-peptide cysteine position** | **Distance (Å)** |
| --- | --- | --- |
|  | **13-13** | **10.28** |
|  | 13-106 | 21.96 |
|  | 13-115 | 17.65 |
|  | 13-117 | 15.83 |
|  | 13-118 | 13.29 |
|  | 106-106 | 31.90 |
|  | 106-115 | 25.99 |
|  | 106-117 | 21.36 |
|  | 106-118 | 21.14 |
|  | 115-115 | 19.80 |
|  | 115-117 | 20.52 |
|  | 115-118 | 14.89 |
|  | **117-177** | **10.20** |
|  | **117-118** | **10.17** |
|  | **118-118** | **10.22** |
