# Supplementary material for: Analysis of the Effects of Polymorphism on Pollen Profilin Structural Functionality and the Generation of Conformational, T- and B-Cell Epitopes
Source: PLoS One. 2013 Oct 17;8(10):e76066. doi: 10.1371/journal.pone.0076066 (PMC3798325; doi:10.1371/journal.pone.0076066)
Supplement: Table S4 — Conservational analysis of residues implicate in PIP-binding domains. A) Residues which percentage of conservation is lower that 100% were highlighted in bold and grey shadowed. Species of Betulaceae and Poaceae genus seem to be the most variable species. B) Examples of punctual changes in the sequence of different profilins from the five species analyzed. Olea europaea L. is the specie with more number of sequences changed. (DOCX) [file pone.0076066.s006.docx]

**Table S4**

**A)**

| **Amino acid and position** | ***Olea europaea*** | ***Betula pendula*** | ***Corylus avellana*** | ***Phleum pratense*** | ***Zea mays*** |
| --- | --- | --- | --- | --- | --- |
| W3 | 100 | 100 | 100 | 100 | 100 |
| D8 | **98.97** | 100 | 100 | 100 | 100 |
| K (71/74) | 100 | 100 | 100 | 100 | 100 |
| Q (76/79) | **95.88** | 100 | 100 | 100 | 100 |
| K (86/89) | 100 | 100 | 100 | 100 | 100 |
| G (88/91) | 100 | 100 | 100 | 100 | 100 |
| M (117/120) | **96.91** | 100 | 100 | 100 | **90** |
| L (127/130) | **87.83** | **0** | **40** | **33.33** | **80** |
| E (128/131) | **93.81** | **0** | **50** | **33.33** | **70** |

**B)**

| **Amino acid and position** | ***Olea europaea* L.** | ***Betula pendula*** | ***Corylus avellana*** | ***Phleum pratense*** | ***Zea mays*** |
| --- | --- | --- | --- | --- | --- |
|  | Sequence accession number | | | | |
| D^8^ → Y | DQ640909 | - | - | - | - |
| Q^79^ → A | DQ138336  DQ640904  DQ640907 | - | - | - | - |
| Q^79^ → R | DQ640905 | - | - | - | - |
| M^117^ → L | - | - | - | - | X73279 |
| M^120^ → I | DQ317564  DQ640908  DQ317577 | - | - | - | - |
| I/L^130^ → D | DQ317567 | - | - | - |  |
| L^130^ → I | DQ138338  DQ317569  DQ138365  DQ117908  DQ138348 | - | - | - | - |
| I/L^130^ → M | DQ663554  DQ663555  DQ663556 | - | - | - | - |
|  |  |  |  |  |  |
| I/L^130^ → V | Y12425  DQ138344  DQ663557 | - | - | - | - |
| L^127^ → A | - |  | DQ663544 | - | - |
| L^127^ → V | - | - | DQ663548 | X77583  Y09456  DQ663536  DQ663541  DQ663542  DQ663562  DQ663563 | X73281  DQ663559 |
| E^128^ → D | - | - | DQ663548  DQ663549  DQ663550 | - | - |
| E^128/131^ → K | DQ663553  DQ663554  DQ663555  DQ663556  DQ663557 | - | DQ663545 | - | - |
| G^130^ → A | - | - | - | - | DQ663565 |
| E^131^ → N | DQ663558 | - | - | - | - |
