# Supplementary material for: Analysis of the Effects of Polymorphism on Pollen Profilin Structural Functionality and the Generation of Conformational, T- and B-Cell Epitopes
Source: PLoS One. 2013 Oct 17;8(10):e76066. doi: 10.1371/journal.pone.0076066 (PMC3798325; doi:10.1371/journal.pone.0076066)
Supplement: Table S5 — Conformational epitopes analysis. The analysis was performed for profilin sequences corresponding to A) Olea europaea L., B) Betula pendula, C) Corylus avellana, D) Phleum pratense, and E) Zea mays. Central residue of conformational epitopes was pointed out with a box. B-cell epitopes partially or totally overlapping with conformational epitopes were highlighted with a color code: red for 10A4, green for 5F2, blue for 9A7, yellow for 9G4, and pink for 3H8. T-cell epitopes partially or totally overlapping with conformational epitopes were highlighted with grey shadows. (DOCX) [file pone.0076066.s007.docx]

**Table S5**

**A)**

| **Specie** | **Allergen** | **Epitope**  **(C)** | **Central residue** | **Sequence** | **Nº of Aas** | **Conserved / Variable residues** |
| --- | --- | --- | --- | --- | --- | --- |
| ***Betula pendula*** | **Bet v 2** | 1 | T5 | **_2_SWQTYVDEHLM_12_** **_37_QSS_39_** | 14 | 14/0 |
|  |  | 2 | S40 | **Q_4_ _8_DEHLM_12_** A_23_ **_37_QSSSFPQ_43_** **I_72_ K_89_ E_109_** | 17 | 16/1 |
|  |  | 3 | Q43 | **_32_GSV_34_** **_37_QSSSFPQFKPQ_4_**_7_ **_68_HLGGI_72_** | 19 | 19/0 |
|  |  | 4 | P46 | **_30_HDGSV_34_** **_42_PQFKPQ_47_** **T_50_ _53_MK_54_** **_68_HLGG_71_** | 19 | 19/0 |
|  |  | 5 | M75 | **_63_APT_65_ H_68_** **_72_IKYMVIQ_78_** **_86_RGKKG_90_** | 16 | 16/0 |
|  |  | 6 | Q78 | **A_63_** **_75_MVIQGE_80_** **_82_GAVIRGK_88_** **P_114_ N_118_** | 16 | 16/0 |
|  |  | 7 | A81 | **_53_MK_54_** **_57_EE**E**GH_61_** **_77_IQGEAGA_83_ R_86_ K_98_** | 16 | 15/1 |
|  |  | 8 | G92 | **_72_IKYM_75_ I_77_ _88_KK_89_ _91_SG_92_ _109_EE_110_** **V_112_ P_114_** | 13 | 13/0 |
|  |  | 9 | P111 | A_23_ **S_91_ _109_EEP_111_VTPG**E_116_ | 10 | 8/2 |

**B)**

| **Specie** | **Allergen** | **Epitope**  **(C)** | **Central residue** | **Sequence** | **Nº of Aas** | **Conserved / Variable residues** |
| --- | --- | --- | --- | --- | --- | --- |
| ***Olea europaea* L.** | **Hev b 8** | 1 | S2 | _2_S**WQ**T**YVDDH_10_** **Q_35_ Y_125_ _129_QGL_131_** | 14 | 13/1 |
|  |  | 2 | R19 | **_8_DDH_10_** **_13_CDI**D**GH**R**LT_21_** _37_SS_38_ _107_D**EP**L**T_111_** | 19 | 13/6 |
|  |  | 3 | S37 + S38 | **Q_4_ D_8_** _19_R**LT_21_ V_32_** **_35_QS**SS**FPQ_41_** **G_69_** D_107_ | 15 | 11/4 |
|  |  | 4 | Q41 | **_30_GSV_32_ _35_QS**SS**FPQFK**SD_45_ **H_66_ _69_GT_70_** | 17 | 13/4 |
|  |  | 5 | S44 + D45 | **_28_HDGSV_32_** **_40_PQFK**SD_45_ _48_AAV**M**K_52_ **T_63_ H_66_ G_69_** | 19 | 14/5 |
|  |  | 6 | T63 | D_45_ _48_AA_49_ K_52_ **_58_G**S**LAPT_63_** **H_66_** **_69_GTKYMV_74_** | 17 | 12/5 |
|  |  | 7 | Q76 | **_57_PG_58_** **A_61_** **_73_MVIQGE**P**GA_81_ _84_RGK_86_** **P_112_ N_116_ E_120_** | 18 | 18/0 |
|  |  | 8 | P79 | **_51_M**K_52_ _55_D**EPG**S_59_ **_74_VIQGE**P**GA_81_** R_84_ K_96_ E_120_ | 18 | 13/6 |
|  |  | 9 | E108 | **_17_GH**R**LT_21_** **_86_KKGS_89_** _107_D**EP_109_**L_110_**TP_112_** | 15 | 12/3 |
|  |  | 10 | M117 | **H_10_ _13_CDI**D**GH_18_** **R_84_ _111_TPG_113_** **_116_NM_117_** **_120_ER_121_** **_124_DY_125_** | 17 | 16/1 |
|  |  | 11 | D128 | **Y_6_ _96_KTGQ_99_** **_120_ER_121_ _124_DYLL**D**QGL_131_** | 15 | 14/1 |
|  | **Bet v 2** | 1 | T5 | **_2_SWQ**T**YVD**E**HLM_12_** **_37_QS**S_39_ | 14 | 11/3 |
|  |  | 2 | S40 | **Q_4_ _8_D**E**HLM_12_** A_23_ **_37_QS**SS**FPQ_43_** I_72_ **K_89_ E_109_** | 16 | 11/5 |
|  |  | 3 | Q43 | **_32_GSV_34_** **_37_QS**SS**FPQFKP**Q_47_ **_68_HLGG**I_72_ | 19 | 15/4 |
|  |  | 4 | P46 | **_30_HDGSV_34_** **_42_PQFKP**Q_47_ T_50_ **_53_M**K_54_ **_68_HLGG_71_** | 19 | 16/3 |
|  |  | 5 | M75 | **_63_APT_65_ H_68_** _72_I**KYMVIQ_78_** **_86_RGKKG_90_** | 16 | 15/1 |
|  |  | 6 | Q78 | **A_63_ _75_MVIQGE_80_** **_82_GAVIRGK_88_ P_114_ N_118_** | 16 | 16/0 |
|  |  | 7 | A81 | **_53_M**K_54_ _57_E**E**E**GH_61_** **_77_IQGEAGA_83_ R_86_ K_98_** | 16 | 13/3 |
|  |  | 8 | G92 | _72_I**KYM_75_** **I_77_ _88_KK_89_** **_91_SG_92_** **_109_EE_110_ V_112_ P_114_** | 13 | 12/1 |
|  |  | 9 | P111 | A_23_ **S_91_** **_109_EE_110_PVTPG**E_116_ | 10 | 8/2 |
|  | **Ara t 8** | 1 | G17 | **_15_VEGN**H_19_ **_107_DEPMT_111_** **Q_114_** | 11 | 10/1 |
|  |  | 2 | A37+K38 | **Q_4_ D_8_ H_19_** T_21_ **V_32_**  **_36_SA**K**FPQ_41_** G_68_ E_70_ **D_107_** | 14 | 10/4 |
|  |  | 3 | Q41 | **G_30_ V_32_** **_36_SA**K**FPQ**L**KP**Q_45_ **_66_FL**GGE_70_ | 17 | 11/6 |
|  |  | 4 | P44+Q45 | **_28_QDG_30_** **_40_PQ_41_ _43_KP**Q_45_ D_48_ _51_K**K_52_** F_59_ **_66_FL**GGE_70_ | 17 | 10/7 |
|  |  | 5 | T63 | **_58_G**F**LAPT_63_** **F_66_** _69_GE**K** **I_75_ K_86_** | 12 | 10/2 |
|  |  | 6 | Q76 | **_57_PG_58_ A_61 75_IQGE**Q_79_ **K_86_ _112_GG_113_** **N_116_** | 12 | 11/1 |
|  |  | 7 | Q79 | _51_K**K_52_** _55_E**EPG**F_59_ **_75_IQG_77_** _79_Q**GA_81_** | 14 | 10/4 |
|  |  | 8 | G88 | _70_E**K_71_** **I_75_ _86_KK_87_** **G_88_** **_107_DE_108_ _111_TG_112_** | 10 | 9/1 |
|  |  | 9 | E108+P109 | **_15_VEGNHL**T_21_ **_86_KKG_88_** **_107_DEPMTG**G**Q_114_** | 18 | 16/2 |
|  |  | 10 | E128+E130 | **W_3_ Q_28_** **W_33_** _98_N**Q_99_** **_120_ER_121_** **_124_DYL**IESE**L_131_** | 15 | 10/5 |

**C)**

| **Specie** | **Allergen** | **Epitope**  **(C)** | **Central residue** | **Sequence** | **Nº of Aas** | **Conserved / Variable residues** |
| --- | --- | --- | --- | --- | --- | --- |
| ***Phleum pratense*** | **Hev b 8** | 1 | S2 | **_2_SWQTYVD**D**H_10_** **Q_35_ Y_125_ _129_QGL_131_** | 14 | 13/1 |
|  |  | 2 | R19 | **_8_D**D**H_10_** **_13_C**D**I**D**GH**R**L**T_21_ _37_SS_38_ **_107_DEP**L**T_111_** | 19 | 11/8 |
|  |  | 3 | S37 + S38 | **Q_4_ D_8_** _19_R**L**T_21_ **V_32_** **_35_QS**SS**FPQ_41_** G_69_ **D_107_** | 15 | 10/5 |
|  |  | 4 | Q41 | **_30_G**SV_32_ **_35_QS**SS**FPQFK**SD_45_ H_66_ _69_GT_70_ | 17 | 9/8 |
|  |  | 5 | S44 + D45 | **_28_HDG**S**V_32_** **_40_PQFK**SD_45_ _48_AAV**MK_52_** **T_63_** H_66_ G_69_ | 19 | 12/7 |
|  |  | 6 | T63 | D_45_ _48_AA_49_ **K_52_** **_58_G**S**LAPT_63_** H_66_ _69_GT**KYMV_74_** | 17 | 10/7 |
|  |  | 7 | Q76 | **_57_PG_58_ A_61_ _73_MVIQGEPGA_81_** **_84_RGK_86_ P_112_ N_116_ E_120_** | 18 | 18/0 |
|  |  | 8 | P79 | **_51_MK_52_** **_55_DEPG**S_59_ **_74_VIQGEPGA_81_** **R_84_ K_96_ E_120_** | 18 | 17/1 |
|  |  | 9 | E108 | **_17_GH**R**L**T_21_ **_86_KKG**S_89_ **_107_DEP**L_110_**TP_112_** | 15 | 11/4 |
|  |  | 10 | M117 | **H_10_ _13_C**D**I**D**GH_18_** **R_84_ _111_TPG_113_** **_116_NM_117_ _120_ER_121_ _124_DY_125_** | 17 | 15/2 |
|  |  | 11 | D128 | **Y_6_ _96_KTGQ_99_** **_120_ER_121_ _124_DYLL**D**QGL_131_** | 15 | 14/1 |
|  | **Ara t 8** | 1 | G17 | _15_V**EG**N**H_19_** **_107_DEPMT_111_** **Q_114_** | 11 | 9/2 |
|  |  | 2 | A37+K38 | **Q_4_** **D_8_ H_19_** T_21_ **V_32_** **_36_SA**K**FPQ_41_** G_68_ E_70_ **D_107_** | 14 | 10/4 |
|  |  | 3 | Q41 | **G_30_ V_32_ _36_SA**K**FPQ**L**KP**Q_45_ **_66_F**LGGE_70_ | 17 | 10/7 |
|  |  | 4 | P44+Q45 | _28_Q**DG_30_** **_40_PQ_41_ _43_KP**Q_45_ D_48_ _51_K**K_52_** F_59_ **_66_F**LGGE_70_ | 17 | 8/9 |
|  |  | 5 | T63 | **_58_G**F**LAPT_63_** **F_66_** _69_GE**K_71_** **I_75_ K_86_** | 12 | 9/3 |
|  |  | 6 | Q76 | **_57_PG_58_ A_61 75_IQGE**Q_79_ **K_86_** _112_G**G_113_** **N_116_** | 12 | 10/2 |
|  |  | 7 | Q79 | _51_K**K_52_**  _55_E**EPG**F_59_ **_75_IQG_77_** _79_Q**GA_81_** | 14 | 10/4 |
|  |  | 8 | G88 | _70_E**K_71_** **I_75_** **_86_KK_87_ G_88_** **_107_DE_108_** **_111_T**G_112_ | 10 | 8/2 |
|  |  | 9 | E108+P109 | _15_V**EG**N**HL**T_21_ **_86_KKG_88_ _107_DEP_109_M_110_T**G**GQ_114_** | 18 | 14/4 |
|  |  | 10 | E128+E130 | Y_3_ Q_28_ W_33_ _98_N**Q_99_** **_120_ER_121_** **_124_DYL**IESE**L_131_** | 15 | 7/8 |

**D)**

| **Specie** | **Allergen** | **Epitope**  **(C)** | **Central residue** | **Sequence** | **Nº of Aas** | **Conserved / Variable residues** |
| --- | --- | --- | --- | --- | --- | --- |
| ***Corylus avellana*** | **Hev b 8** | 1 | S2 | **_2_SWQTYVD**D**H_10_** **Q_35_ Y_125_ _129_QGL_131_** | 4 | 14/0 |
|  |  | 2 | R19 | **_8_D**D**H_10_** **_13_C**D**IDGH**R**LT_21_** **_37_S**S_38_ **_107_DEPLT_111_** | 19 | 15/4 |
|  |  | 3 | S37 + S38 | **Q_4_ D_8_** _19_R**L**T_21_ **V_32_ _35_QSS**S**FPQ_41_ G_69_ D_107_** | 15 | 12/3 |
|  |  | 4 | Q41 | **_30_GSV_32_** **_35_QSS**S**FPQFK**SD_45_ **H_66_ _69_G**T_70_ | 17 | 13/4 |
|  |  | 5 | S44 + D45 | **_28_HDGSV_32_ _40_PQFK**SD_45_ **_48_AA**VM**K_52_** **T_63_ H_66_ G_69_** | 19 | 15/4 |
|  |  | 6 | T63 | **D_45_** **_48_AA_49_** **K_52_ _58_GSLAPT_63_** **H_66_ _69_G**T**KYMV_74_** | 17 | 16/1 |
|  |  | 7 | Q76 | **_57_PG_58_ A_61_ _73_MVIQGE**P**GA_81_** **_84_RGK_86_ P_112_ N_116_ E_120_** | 18 | 17/1 |
|  |  | 8 | P79 | **_51_MK_52_** **_55_DEPGS_59_ _74_VIQGE**P**GA_81_** **R_84_ K_96_ E_120_** | 18 | 17/1 |
|  |  | 9 | E108 | **_17_GHRLT_21_** **_86_KKGS_89_** **_107_DEP_109_L_110_TP_112_** | 15 | 15/0 |
|  |  | 10 | M117 | **H_10_ _13_CDIDGH_18_ R_84_ _111_TPG_113_ _116_NM_117_ _120_ER_121_ _124_DY_125_** | 17 | 17/0 |
|  |  | 11 | D128 | **Y_6_ _96_KT**G**Q_99_** **_120_ER_121_ _124_DYLL**D**QGL_131_** | 15 | 13/2 |
|  | **Bet v 2** | 1 | T5 | **_2_SWQTYVDEHLM_12 37_QSS_39_** | 14 | 14/0 |
|  |  | 2 | S40 | **Q_4_ _8_DEHLM_12_ A_23_ _37_QSSSFPQ_43_** I_72_ **K_89_ E_109_** | 17 | 16/1 |
|  |  | 3 | Q43 | **_32_GSV_34_ _37_QSSSFPQFKPQ_47 68_HLGGI_72_** | 19 | 0/0 |
|  |  | 4 | P46 | **_30_HDGSV_34_** **_42_PQ**F**KP**Q_47_ **T_50_ _53_MK_54_ _68_HLGG_71_** | 19 | 17/2 |
|  |  | 5 | M75 | **_63_APT_65_ H_68_ _72_**I**KYMVIQ_78_** **_86_RGKKG_90_** | 16 | 15/1 |
|  |  | 6 | Q78 | **A_63_ _75_MVIQGE_80_** **_82_GAVIRGK_88_ P_114_ N_118_** | 16 | 16/0 |
|  |  | 7 | A81 | **_53_MK_54_** _57_E**E**E**GH_61_ _77_IQGEAGA_83_** **R_86_ K_98_** | 16 | 14/2 |
|  |  | 8 | G92 | _72_I**KYM_75_** **I_77_ _88_KK_89_ _91_SG_92_** **_109_EE_110_ V_112_ P_114_** | 13 | 12/1 |
|  |  | 9 | P111 | **A_23_ S_91_ _109_EEPVTPG**E_116_ | 10 | 9/1 |
|  | **Ara t 8** | 1 | G17 | **_15_VEGNH_19_ _107_DEPMT_111_ Q_114_** | 11 | 11/0 |
|  |  | 2 | A37+K38 | **Q_4_ D_8_ H_19_ T_21_ V_32_** **_36_SA**K**FPQ_41_** G_68_ E_70_ **D_107_** | 14 | 11/3 |
|  |  | 3 | Q41 | **G_30_ V_32_ _36_SA**K**FPQLKPQ_45_** **_66_FL**GGE_70_ | 17 | 14/3 |
|  |  | 4 | P44+Q45 | **_28_QDG_30_ _40_PQ_41_ _43_KPQ_45_** D_48_ **_51_KK_52_** F_59_ **_66_FL**GGE_70_ | 17 | 12/5 |
|  |  | 5 | T63 | **_58_G**F**LAPT_63_** **F_66_** _69_GE**K I_75_** **K_86_** | 12 | 10/2 |
|  |  | 6 | Q76 | **_57_PG_58_ A_61_** **_75_IQGE**Q_79_ **K_86_** _112_G**G_113_** **N_116_** | 12 | 10/2 |
|  |  | 7 | Q79 | _51_K**K_52_** **_55_EEPG**F_59_ **_75_IQG_77_** _79_Q**G**A_81_ | 14 | 10/4 |
|  |  | 8 | G88 | _70_E**K_71_ I_75_ _86_KK_87_** **G_88_** **_107_DE_108_ _111_T**G_112_ | 10 | 8/2 |
|  |  | 9 | E108+P109 | **_15_VEGNHLT_21_** **_86_KKG_88_ _107_DEP_109_M_110_T**G**GQ_114_** | 18 | 17/1 |
|  |  | 10 | E128+E130 | **W_3_ Q_28_ W_33_** _98_N**Q_99_** _120_E**R_121_** **_124_DYLIE**SE**L_131_** | 15 | 11/4 |

**E)**

| **Specie** | **Allergen** | **Epitope**  **(C)** | **Central residue** | **Sequence** | **Nº of Aas** | **Conserved / Variable residues** |
| --- | --- | --- | --- | --- | --- | --- |
| ***Zea mays*** | **Hev b 8** | 1 | S2 | **_2_SWQ**T**YVD**D**H_10_** **Q_35_ Y_125_ _129_QGL_131_** | 14 | 13/1 |
|  |  | 2 | R19 | **_8_D**D**H_10_** **_13_C**D**I**D**GH**R**LT_21_** _37_SS_38_ **_107_DEP**L**T_111_** | 19 | 13/6 |
|  |  | 3 | S37 + S38 | **Q_4_ D_8_** _19_R**LT_21_** V_32_ **_35_QS**SS**FPQ_41_** G_69_ **D_107_** | 15 | 10/5 |
|  |  | 4 | Q41 | **_30_G**S**V_32_** _35_QSSS**FPQFK**SD_45_ H_66_ _69_G**T_70_** | 17 | 8/9 |
|  |  | 5 | S44 + D45 | **_28_HDG**SV_32_ **_40_PQFK**SD_45_ **_48_A**AV**MK_52_** **T_63_** H_66_ G_69_ | 19 | 11/8 |
|  |  | 6 | T63 | D_45_ **_48_A**A_49_ **K_52_** **_58_G**S**LAPT_63_** H_66_ _69_G**TKYMV_74_** | 17 | 12/5 |
|  |  | 7 | Q76 | **_57_PG_58_ A_61_** **_73_MVIQGEPGA_81_** **_84_RGK_86_ P_112_ N_116_ E_120_** | 18 | 18/0 |
|  |  | 8 | P79 | **_51_MK_52_** **_55_DEPG**S_59_ **_74_VIQGEPGA_81_** **R_84_** **K_96_ E_120_** | 18 | 17/1 |
|  |  | 9 | E108 | **_17_GH**R**LT_21_ _86_KKGS_89_ _107_DEP_109_**L_110_**TP_112_** | 15 | 13/2 |
|  |  | 10 | M117 | **H_10_ _13_C**DID**GH_18_** **R_84_ _111_TPG_113_** **_116_NM_117_ _120_ER_121_ _124_DY_125_** | 17 | 14/3 |
|  |  | 11 | D128 | **Y_6_ _96_KTGQ_99_ _120_ER_121_ _124_DYLL**D**QGL_131_** | 15 | 14/1 |
